# Nutraceutical potential of radish (*Raphanus sativus* cv. Tango) microgreens as sustainable and reproducible sources of hydrogen sulfide-releasing compounds

**DOI:** 10.1016/j.fochx.2026.103816

**Published:** 2026-04-02

**Authors:** Maria Maisto, Adua Marzocchi, Vincenzo Piccolo, Erika Esposito, Melania Correale, Emma Mitidieri, Gian Carlo Tenore, Roberta d'Emmanuele di Villa Bianca

**Affiliations:** a Nutrapharmalab, Department of Pharmacy, School of Medicine and Surgery, University of Napoli Federico II, Via Domenico Montesano 49, 80131 Napoli, Italy; b Department of Pharmacy, School of Medicine and Surgery, University of Napoli Federico II, Via Domenico Montesano 49, 80131 Napoli, Italy

**Keywords:** Glucosinolates, Radish microgreens, Hydrogen sulfide, Sulfanutraceuticals

## Abstract

Hydrogen sulfide (H_2_S) is a key endogenous gasotransmitter involved in vascular homeostasis. Among dietary phytochemicals, glucosinolates (GSLs) from *Brassicaceae* represent natural H_2_S-releasing compounds with nutraceutical potential. This study introduces *Raphanus sativus* cv. Tango microgreens, grown through a fully controlled soilless hydroponic system, as a standardized and sustainable bioplatform for GSLs production. An HPLC-qQq-MS/MS method was validated for the accurate identification and quantification of intact GSLs, and cultivation conditions were optimized to maximize their accumulation. The optimized microgreens sample enriched in GSLs (70.71 mg/g DW), particularly glucoraphasatin, also displayed a valuable polyphenolic profile associated with relevant antioxidant *in vitro* activity. The optimized extract showed concentration-dependent H_2_S-releasing activity in both cell-free conditions and bovine aortic endothelial cells. In an *in vitro* model of endothelial lipid overload, it reduced ROS production and improved nitric oxide bioavailability, showing a protective antioxidant profile. These findings support radish microgreens as promising sulfanutraceuticals for cardiovascular health management.

## Highlights

• Red-light hydroponic growth maximized GSLs in radish microgreens at day 6.
• Precursor ion scan profiling enabled comprehensive detection of minor glucosinolates.
• Validated HPLC–qQq-MS/MS enabled highly sensitive quantification of intact GSLs.
• Optimized extract showed strong antioxidant and concentration-dependent H₂S release.

## Graphical abstract

**Figure:**
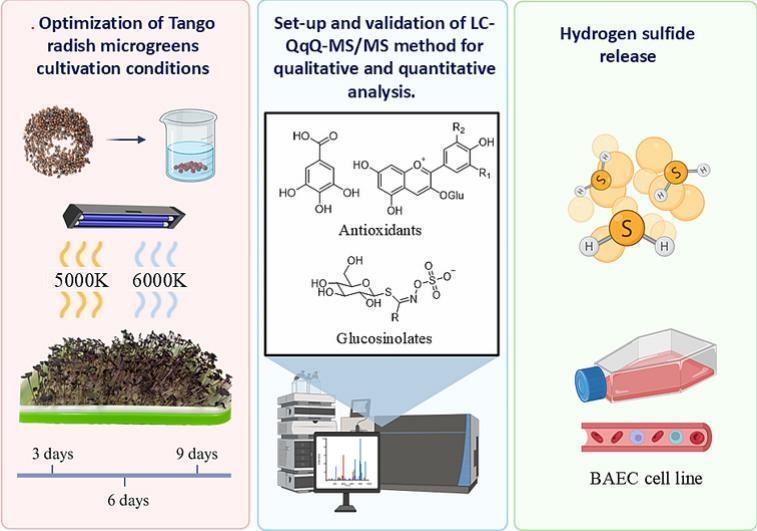

## 1 Introduction

Hydrogen sulfide (H_2_S) was historically known primarily for its pungent, rotten-egg odor and toxicity upon inhalation. In 1996, however, Abe and Kimura first demonstrated that H_2_S is endogenously produced within the mammalian central nervous system (Abe & Kimura, 1996). This discovery marked the beginning of a new era in the pharmacological investigation of sulfur-containing compounds. Today; H_2_S is recognized as the third endogenous gasotransmitter; alongside nitric oxide (NO) and carbon monoxide (CO); playing a key role in maintaining organ and tissue function. H_2_S-producing enzymes are expressed in all organs and play an important role in their physiology. Specifically; H_2_S is a critical regulator of vascular homeostasis; influencing processes such as endothelial function and vascular tone (Cirino et al., 2023; Citi et al., 2021). The chronic pro-inflammatory state and elevated oxidative stress accelerate vascular damage, leading to impaired endothelial function and increased cardiovascular risk. Notably, H_2_S exerts several beneficial effects through acting on various targeted signaling pathways implicated in cardiovascular disease associated with endothelial dysfunction. At the cardiovascular level, it enhances endothelial function, attenuates vascular inflammation and oxidative stress, restores redox balance, and upregulates endogenous NO synthesis (Bello et al., 2023; d'Emmanuele di Villa Bianca et al., 2015; Mitidieri et al., 2020; Piccolo, Maisto, Cerrato, et al., 2024). Specifically, H₂S acts as an endogenous antioxidant signaling molecule playing a crucial role in protecting cells from oxidative stress. It acts directly as scavenger of reactive oxygen species (ROS) and enhances the activity of antioxidant enzymes. In addition, H₂S supports cellular redox balance by maintaining intracellular glutathione (GSH) levels and strengths antioxidant defenses through activation of the Nrf2 signaling pathway (Yarmohammadi et al., 2021; Zhang et al., 2024). The physiological relevance of H_2_S is further underscored by clinical evidence reporting significantly reduced plasma H₂S levels in pathological conditions such as diabetes, hypertension, and obesity, in both animal models and human (Jain et al., 2010; Piragine et al., 2023). This evidence stimulated growing interest in sulfanutraceutics, defined as edible or drinkable complex matrices containing one or more H₂S-releasing sulfur compounds, as promising nutraceutical tools for the prevention and management of cardiovascular-related disorders (Martelli et al., 2023a). Among these; glucosinolates (GSLs); a class of sulfur-containing phytochemicals predominantly found in *Brassicaceae* plants such as broccoli; kale; and radishes; have recently emerged as promising candidates. These compounds; produced by plants as defense molecules; are biosynthesized from α-amino acids and share a conserved structure comprising four main moieties: a glucose residue; an (*Z*)-thiohydroximate group; a sulfate group; and a variable side chain derived from the amino acid precursor (Bones & Rossiter, 1996). In plant tissues; GSLs are stored in specialized cells (like S-cells); while the myrosinase enzyme (β-thioglucosidase glucohydrolase; EC 3.2.1.147) is compartmentalized in separate myrosin cells. Upon tissue damage; these components come into contact; myrosinase hydrolyzes GSLs to yield bioactive products; mainly isothiocyanates (ITCs); which can release H₂S through reactions with nucleophilic agents (Martelli et al., 2020). In addition; recent evidence suggests that GSLs may exert biological effects through their intrinsic H_2_S-releasing potential; independently of myrosinase-mediated conversion to ITCs (Gambari et al., 2022; Piccolo, Maisto, Cerrato, et al., 2024).

Despite this potential, the efficient recovery of intact GSLs from plant sources remains challenging due to their susceptibility to enzymatic degradation during the extraction process. In particular, inadequate processing conditions can activate endogenous myrosinase, triggering rapid hydrolysis of GSLs and leading to their structural degradation and reduced yields. To address this, several extraction strategies have been proposed to inactivate myrosinase activity, most commonly concerning thermal treatments or lyophilization, applied before or during extraction procedures. Nevertheless, the overall yield and qualitative profile of GSLs are additionally strongly influenced by multiple factors, including the plant species and cultivar, anatomical part used, developmental stage, soil characteristics, climate, growing season, water availability, pathogen exposure, and postharvest conditions (Liu et al., 2022).

To overcome these limitations, plant “biofactories” have been introduced as sustainable and controllable platforms for the production of natural bioactive compounds (López-Belchí et al., 2024). Currently; several biofactory systems have been developed; including callus cultures; cell suspension cultures; and; more recently; microgreens (Laezza et al., 2024). Specifically; microgreens are edible seedlings harvested 1–3 weeks after germination and are characterized by high concentrations of bioactive phytochemicals (Fayezizadeh et al., 2024). Compared to callus or suspension cultures; microgreens offer several advantages: rapid growth cycles; low production costs; and compatibility with hydroponic cultivation; which optimizes nutrient delivery; reduces water use by up to 90% compared with conventional soil-based systems; and enables vertical farming (Galieni et al., 2020; Naresh et al., 2024). Moreover, their phytochemical content can be modulated through elicitors of chemical, physical, or biological nature, providing further opportunities to enhance secondary metabolite production (Galieni et al., 2020). In this context; *Brassicaceae* microgreens such as radish; cabbage; and rocket have recently been explored as controlled and reproducible sources of GSLs. In addition; recent studies have highlighted the value of combining robust analytical characterization with functional *in vitro* evaluation to support the biological and applicative potential of natural extracts and plant-derived matrices (Di Lorenzo et al., 2021, Di Lorenzo et al., 2024).

In light of these considerations, the main objective of the present study is the optimization of the growth conditions for *Raphanus sativus* cv. Tango microgreens, to maximize the quantitative accumulation of GSLs in the edible biomass. To accurately determine the specific GSL profile and concentration, a specific HPLC-qQq-MS/MS analytical method was developed and validated. In parallel, a standardized extraction protocol was established to preserve GSL integrity and ensure reproducibility. Finally, preliminary assessments of the H_2_S-releasing capacity of extracts obtained from the microgreen sample with maximized GSL concentration (OME, optimized microgreen extract) will be conducted using both a cell-free system and an *in vitro* cellular model, employing bovine aortic endothelial cells (BAECs). Since the functional investigation of *Brassicaceae*-derived microgreens extract are scarly reported, this work represents the first attempt to explore the potential of microgreens-based extract to modulate the H₂S metabolism and its downstream effects on endothelial redox homeostasis by *in vitro* models.

## 2 Materials and methods

### 2.1 Reagents

All chemicals, reagents, and standards used were analytical or LC-MS grade reagents. Glucoraphanin potassium salt (purity ≥99% HPLC), glucoraphasatin potassium salt (purity ≥95% HPLC), and glucoraphenin potassium salt (purity ≥99% HPLC) were purchased from Phytolab (Vestenbergsgreuth, Germany). Gallic acid (≥ 98%), Folin–Ciocalteu reagent (≥ 99.9%), 2,2-diphenyl-1-picrylhydrazyl (DPPH) (≥ 99.9%), 6-hydroxy-2,5,7,8-tetramethylchroman-2-carboxylic acid (Trolox) (≥ 99.9%), Sodium carbonate (Na_2_CO_3_), HPLC- and LC-MS-grade methanol (≥ 99.9%), and Thioglucosidase from *Sinapis alba* (white mustard) seed (Batch number BCCM8294, enzymatic activity 558.4 U/g) were acquired from Sigma-Aldrich (Milan, Italy). Formic acid (98–100%) were supplied from Romil Ltd. (Cambridge, UK). Water was obtained by a Milli-Q water purification system (Millipore, Bedford, MA, USA).

### 2.2 Plant material

Seeds of the radish cultivar ‘Tango’ (*Raphanus sativus* L. cv. Tango) were purchased from a local supplier. The germination rate was determined to be 99%. A total of 5 g of seeds were soaked in tap water for 12 h to promote germination. Afterward, the seeds were evenly spread across the surface of seedling trays at a rate of 5 g per tray. The trays used consisted of two parts: a mesh seed tray and a base tray for holding water, each measuring 33 cm in length, 24 cm in width, and 4 cm in depth. Following seed distribution, 0.6 L of water was added to the lower tray, just enough to reach the base of the mesh tray containing the seeds. The seeds were allowed to germinate in complete darkness at a temperature of 25 ± 0.2 °C for three days. After germination, the seedlings were cultivated hydroponically in the same tray setup, with roots suspended in clean water enriched with a nutrient solution with the following average concentrations [g/L]: N, 60; P₂O₅, 60; K₂O, 60; B, 0.5; Cu, 0.1; Mn, 0.1; Zn, 0.1. The solution was refreshed daily to maintain water quality. The trays were positioned in a vertically stacked multilayer growth chamber using a completely randomized design with three replicates and divided into two lighting treatments. Two experimental groups were grown under red light-emitting diodes (LED) light (630–650 nm, RL) and blue LED light (460–465 nm, BL), respectively (Philips GreenPower LED production modules; Koninklijke Philips Electronics, The Netherlands). The LED source was maintained at a vertical distance of 40 cm from the seed tray surface, providing a photosynthetic photon flux density (PPFD) of 150 μmol m^−2^ s^−1^ at canopy level, with plants receiving 16 h of light daylily (Ying et al., 2020). Microgreens were harvested at three growth stages: the 3rd, 6th, and 9th days after germination. Harvesting involved cutting the seedlings just above the mesh surface. The collected microgreens were stored at −80 °C prior to freeze-drying. Once dried, the samples were ground into powder and stored at −20 °C until further analysis.

### 2.3 Glucosinolates extraction procedure

The extraction of GSLs from radish microgreens was carried out following a previously established method, with slight modifications (Piccolo, Maisto, Cerrato, et al., 2024). Specifically, 10 mL of a 1% formic acid aqueous solution, preheated to 75 °C, was added to 1 g of the sample matrix. The samples underwent sonication for 10 min using an ultrasonic bath (Branson Fisher Scientific 150 E Sonic Dismembrator) and were subsequently shaken on an orbital shaker (Sko-DXL, Argolab, Carpi, Italy) at 600 rpm for 10 min at 75 °C. Afterwards, the samples were centrifuged at 9000 rpm for 15 min. The resulting supernatants were collected and stored at 4 °C, protected from light exposure. The remaining pellets were subjected to a second extraction using 10 mL of the same extraction solution and procedure. The supernatants obtained from the second extraction step were combined with those from the first and then frozen before lyophilization. For the HPLC-MS/MS analysis, the dried extracts were prepared at concentrations of 0.1 and 1 mg/mL. The lyophilized samples were dissolved in an aqueous solution containing 20% methanol. Following dissolution, the samples were filtered through a 0.22 μm nylon membrane filter (Cell Treat, Shirley, MA, USA) before HPLC-MS/MS analysis. The results are expressed as mg of each GSL/g of dry weight (DW) of radish microgreen. The same batch of extracts were used for all subsequent analyses, including GSLs quantification, antioxidant assays, and H₂S-releasing activity, to ensure consistency and comparability of the results.

### 2.4 Evaluation of myrosinase thermal inactivation

Myrosinase activity was evaluated at different temperatures (25 °C. 45 °C, 55 °C, 65 °C, 75 °C) according to a procedure adapted from (Pardini et al., 2021), using glucoraphasatin as substrate. The reaction mixture was prepared by combining 250 μL of myrosinase solution (0.5 U/mL), 250 μL of glucoraphasatin solution (0.1 mg/mL), and 500 μL of phosphate buffer (50 mM, pH 6.0). Both myrosinase and glucoraphasatin solutions were prepared by dissolving the respective standards in phosphate buffer 50 mM, pH 6.0. The mixture was incubated at the selected temperatures (25–75 °C), and aliquots of 100 μL were collected at predetermined time hour intervals (0, 1, 2, 3, 4). Each aliquot was immediately quenched by the addition of 100 μL of methanol at 0.1% of formic acid to stop the enzymatic reaction. The samples were then centrifuged at 13.000 rpm for 10 min at room temperature. The supernatants were collected and analyzed by HPLC-qQq-MS/MS to determine glucoraphasatin hydrolysis (%). The hydrolysis percentage was calculated relative to a control sample in which the enzyme solution was replaced by phosphate buffer. Individual stock solutions of glucoraphasatin and myrosinase were prepared in phosphate buffer 50 mM, pH 6.0 at a concentration of 1 mg/mL and 1 U/mL, respectively. The stock solutions were diluted in phosphate buffer 50 mM, pH 6.0 to obtain the concentrations to be tested. The samples were analyzed in triplicate.

### 2.5 Intact GSLs quantification by HPLC-qQq-MS/MS analysis

An Agilent 1200 HPLC, coupled with an Agilent Technologies 6470 triple quadrupole mass spectrometer (Agilent Technologies, Palo Alto, CA, USA), was used for the analysis. The analysis was performed according to Piccolo, Maisto, Cerrato, et al. (2024), with slight modifications. Separation was performed using as column a Kinetex® XB-C18 column (50 mm × 3 mm, 2.6 μm, Phenomenex, Torrance, CA, USA). The analysis was performed using a heated electrospray ionization (HESI) source operated in negative ion mode, with multiple reaction monitoring (MRM) scanning mode employed. In this mode, one transition was selected as the quantifier and a second as the qualifier. MRM transitions, collision energy (CID), and fragmentor values are summarized in Supplementary Materials, Table S1. Argon was used as gas for collision-induced fragmentation. The MRM parameters were optimized using an aqueous solution at 20% methanol of each analytical standard at a concentration of 10 ppm. The ion source was set using the following parameters: gas flow rate: 7 L/min; gas temperature: 180 °C; sheath gas flow rate: 11 L/min; sheath gas temperature: 350 °C; nebulizer pressure: 45 psi; capillary voltage: 2500 V; nozzle voltage: 2000 V.

### 2.6 Qualitative analysis of intact GSLs by HPLC-qQq-MS/MS analysis

The qualitative characterization of intact GSLs was carried out using an Agilent 1200 HPLC system coupled to an Agilent Technologies 6470 triple quadrupole mass spectrometer (Agilent Technologies, Palo Alto, CA, USA). Chromatographic separation was achieved with the method reported by Piccolo, Maisto, Cerrato, et al. (2024), with slight modifications. Separation was performed using a Kinetex® XB-C18 column (50 mm × 3 mm, 2.6 μm, Phenomenex, Torrance, CA, USA). The mobile phases consisted of water containing 0.01% of formic acid (solvent A) and methanol containing 0.01% of formic acid (solvent B). The elution started with an isocratic step at 2% B for 2 min, followed by a linear increase to 45% B at 12 min and to 95% B at 14 min, which was maintained for 2 min. The column was then re-equilibrated at initial conditions for 2 min. Analyses were performed with an injection volume of 5 μL, a column temperature of 35 °C, and a flow rate of 0.5 mL/min. Mass spectrometric detection was performed using a heated electrospray ionization (HESI) source operating in negative ion mode. Data were acquired in full scan (FS), tandem MS (MS/MS), and precursor ion scan (PIS) acquisition modes. The setup of the PIS protocol was established through a preliminary evaluation conducted on a panel of 14 GSLs analytical standards (*e.g.*, glucoraphanin, glucoraphenin, glucoraphasatin, sinigrin, sinalbin, gluconapin, glucobrassicanapin, progoitrin, epiprogoitrin, glucoalyssin, glucobrassicin, 4-hydroxyglucobrassicin, neoglucobrassicin, 4-methoxyglucobrassicin). Based on the fragmentation patterns observed, specific precursor ions and optimal collision energies were selected for the analysis. Precursor ion scans targeting the ions with *m/z* 259, *m/z* 97, and *m/z* 96 were performed using collision energies of 15, 20, and 40 eV, respectively. Product ion spectra were subsequently acquired at a collision energy of 20 eV. Ion source parameters were maintained identical to those employed for quantitative GSL analysis.

### 2.7 Analytical validation of HPLC-qQq-MS/MS analysis

Method validation was conducted in accordance with the ICH validation guideline (ICH.Q2[R1], 1995) (Ohno, 2002), including the assessment of linearity, sensitivity (LOD and LOQ), precision, accuracy, analytical stability, and carry-over. Individual stock solutions of GSLs standards were prepared in water at a concentration of 1 mg/mL and subsequently combined to obtain a mixed standard solution at 100 ppm in 20% aqueous methanol. Calibration curves were generated by serial dilution (1,2) of the mixed standard solution and were analyzed in triplicate at each concentration level. Linearity was evaluated by least-squares regression analysis of peak area *versus* analyte concentration, yielding correlation coefficients (R^2^ ≥ 0.99). Calibration ranges comprised 12 concentration levels (5 ppm–2.5 ppb) for glucoraphanin and glucoraphenin and 10 levels (5 ppm–10 ppb) for glucoraphasatin. Method sensitivity was estimated by determining the limits of detection and quantification based on signal-to-noise ratios of 3 and 10, respectively, calculated from low-concentration standards compared with blank injections. Accuracy and precision were evaluated through intra-day and inter-day analyses performed at three concentration levels for each analyte. Intra-day repeatability was assessed by three replicate injections within the same day, whereas inter-day precision was determined over three consecutive days. Accuracy was expressed as percentage bias, while precision was reported as coefficient of variation (CV%).

To monitor analytical stability throughout the HPLC-MS/MS sequence, quality control (QC) samples were prepared using a 5 ppm mixed standard solution of the three GSLs. QC samples were injected at regular intervals (every 8–10 analytical samples) across the sequence. For each QC injection, peak area and retention time were evaluated. Analytical performance was considered acceptable if the relative standard deviation (RSD%) of the peak area for each compound did not exceed 5%, and if retention time variation remained within ±0.2 min.

To assess the potential carry-over in the HPLC-MS/MS system, a sequence of three injections of a high-concentration standard solution of each compound at a concentration of 5 ppm was followed by three injections of a low-concentration solution, corresponding to the lowest calibration point of the respective standard curve for each compound. The carry-over effect was calculated as follows:

$$\text{Carry}-\text{over}\;\left(\%\right)=\frac{L1-\mathrm{mean}\left(L2,L3\right)}{H3-\mathrm{mean}\left(L2,L3\right)}*100$$

where L1 is the first injection of low concentration solution, L2 is the second injection of low concentration solution, L3 is the third injection of low concentration solution, and H3 is the third injection of high concentration solution.

### 2.8 Polyphenols and anthocyanin extraction procedure

The extraction of polyphenols and anthocyanins from selected radish microgreens with maximized GSLs concentration was carried out following a previously reported method with slight adjustments (Maisto et al., 2023). In detail, 0.5 g of freeze-dried sample was combined with 5 mL of a solution composed of 80% methanol in water containing 1% formic acid. The mixture was vortexed for 1 min to ensure proper mixing. Subsequently, the samples were subjected to ultrasonic treatment in a bath (Branson Fisher Scientific 150 E Sonic Dismembrator) for 10 min, followed by shaking on an orbital shaker (Sko-DXL, Argolab, Carpi, Italy) at 600 rpm for another 10 min. After this, centrifugation was performed at 9000 rpm for 10 min. The resulting supernatants were carefully collected and stored at 4 °C, protected from light. The residual pellets were then subjected to a second extraction using the same procedure and solvent volume. The combined extracts were stored at −20 °C until further HPLC-HESI-MS/MS analysis.

### 2.9 Qualitative analysis of polyphenols and anthocyanins by HPLC-HESI-MS/MS analysis

Qualitative profiling of polyphenolic compounds and anthocyanins was performed using an UltiMate 3000 HPLC system (Thermo Fisher Scientific, San Jose, CA, USA) coupled to an LTQ XL ion trap mass spectrometer equipped with a heated electrospray ionization (HESI) source. Chromatographic separation was achieved with the method reported by (Piccolo, Maisto, Schiano, et al., 2024), with slight modifications. Separation was performed using a Kinetex® C18 column (75 mm × 2.1 mm, 2.6 μm; Phenomenex, Torrance, CA, USA). The mobile phases consisted of water containing 0.1% of formic acid (solvent A) and acetonitrile containing 0.01% of formic acid (solvent B). The elution started with an isocratic step at 5% B for 1.5 min, followed by a linear increase to 95% B at 36 min, which was maintained for 5 min. The column was then re-equilibrated at initial conditions for 4 min. Analyses were performed with an injection volume of 5 μL, a column temperature of 35 °C, and a flow rate of 0.35 mL/min. Mass spectrometric acquisition was carried out in both positive and negative ionization modes using full scan (FS) and data-dependent acquisition (DDA) experiments. Fragmentation was induced by collision-induced dissociation using argon as collision gas and a normalized collision energy of 35 eV. The ion source in positive acquisition mode was set using the following parameters: sheath gas flow rate: 30 mL/min; auxiliary gas flow rate: 10 mL/min; capillary temperature: 320 °C; source heated temperature: 150 °C; source voltage: 3.5 kV; source current: 100.0 μA; capillary voltage: 32.0 V; tube lens: 80.0 V. The ion source in negative acquisition mode was set using the following parameters: sheath gas flow rate: 30 mL/min; auxiliary gas flow rate: 10 mL/min; capillary temperature: 320 °C; source heated temperature: 150 °C; source voltage: 3.1 kV; source current: 100.0 μA; capillary voltage: −43.0 V; tube lens: −166.41 V.

### 2.10 Total polyphenols content (TPC)

The total phenolic compounds were quantified in the samples using the Folin–Ciocalteu colorimetric method, as described by (Piccolo, Maisto, Schiano, et al., 2024). The reaction was initiated by combining 0.125 mL of hydroalcoholic extract with 0.125 mL of Folin–Ciocalteu reagent, 1.25 mL of a 7.5% (w/v) aqueous Na₂CO₃ solution, and 1.5 mL of distilled water to reach a final volume of 3 mL. After mixing for 10 s, the reaction mixtures were incubated in the dark for 90 min. Following incubation, absorbance was measured at 760 nm using a V-730 UV–Visible/NIR spectrophotometer operated with the Spectra Manager™ Suite software (Jasco Inc., Easton, MD, USA). Gallic acid was used to create the calibration curve, which included six concentrations ranging from 7.5 to 250 ppm. Each calibration point was measured in triplicate using a 1:2 dilution factor. All sample analyses were conducted in triplicate, and the results were expressed as mg gallic acid equivalents (GAE)/g DW.

### 2.11 Total anthocyanins content (TAC)

The total anthocyanin content (TAC) was determined using a modified version of the pH-differential spectrophotometric method described by Swer et al. (2016). For each sample, two dilutions were prepared. One dilution was made with potassium chloride buffer at pH 1.0 (2.5 mM), while the other was made with sodium acetate buffer at pH 4.5 (0.4 M). After incubating for 15 min, the absorbance of each dilution was recorded at 510 nm and 700 nm against distilled water as a blank. The TAC was calculated using the following formula:

$$\mathrm{TAC}\;\left(\mathrm{mg}/L\right)=\frac{\left[\left(A520-A700\right)\mathrm{pH}1.0-\left(A520-A700\right)\mathrm{pH}4.5\right]*\mathrm{MW}*\mathrm{DF}*1000\;}{\varepsilon *L}$$

where TAC is the total anthocyanins content expressed as cyanidin-3-glucoside equivalent (CGE) (mg/L), using the molecular weight (MW) of cyanidin-3-glucoside (449.2 g/mol), the molar absorbance ε of cyanidin-3-glucoside (26,900), a cell path length L of 1 cm and a dilution factor DF. The results were reported as mg of cyanidin-3-glucoside equivalents per gram of microgreen dry weight (mg CGE/g DW).

### 2.12 Antioxidant activity

#### 2.12.1 DPPH assay

The antioxidant capacity of the samples was assessed using the DPPH (2,2-diphenyl-1-picrylhydrazyl) radical scavenging assay, as previously outlined (Iannuzzo et al., 2022). In accordance with the standard procedure, 200 μL of hydroalcoholic extract was added to 1000 μL of a 0.05 mM DPPH methanolic solution, and the mixture was incubated in the dark for 10 min. After the reaction time, the reduction in absorbance was recorded at 517 nm using a V-730 UV–Visible/NIR spectrophotometer with Spectra Manager™ Suite software (Jasco Inc., Easton, MD, USA). The absorbance of the DPPH solution without extract served as the blank. The percentage of inhibition was calculated using the equation:

$$\%\text{inhibition}=\frac{1-\mathrm{Af}}{\mathrm{Ac}}*100$$

where Af is the absorbance after 10 min, and Ac is the absorbance of the control at time zero. 6-hydroxy-2,5,7,8-tetramethylchroman-2-carboxylic acid (Trolox) was used as reference antioxidant, and a calibration curve was generated using eight concentrations ranging from 5 to 250 μM. Each concentration was tested in triplicate using dilution factors of 1:2 and 1:5. Extracts were analyzed in triplicate, and results were expressed as μmol Trolox equivalent (TE)/g DW. Additionally, IC₅₀ values were calculated by testing extract concentrations between 0.01 and 10 mg/mL.

#### 2.12.2 ABTS assay

The ABTS assay was employed to assess the ability of the extracts to quench ABTS (2,2′-azino-bis-(3-ethylbenzothiazoline-6-sulfonic acid)) radical, based on the method reported by Iannuzzo et al. (2022). The ABTS working solution was prepared by mixing 2.5 mL of 7.0 mM ABTS with 0.044 mL of 140 mM potassium persulfate. This solution was incubated in the dark at 5 °C for 7 h. Subsequently, the solution was diluted with ethanol until an absorbance of 0.70 ± 0.05 at 754 nm was achieved (Jasco Inc., Easton, MD, USA). For the assay, 0.10 mL of the hydroalcoholic extract was mixed with 1 mL of the ABTS ethanolic solution and incubated in the dark for 2.5 min. The reduction in absorbance was then recorded at 517 nm. The ethanol-diluted ABTS solution without a sample was used as the blank. Inhibition percentage was determined using the formula:

$$\%\text{inhibition}=\frac{1-\mathrm{Af}}{\mathrm{Ac}}*100$$

where Af is the absorbance after 10 min, and Ac is the absorbance of the control at time zero. Trolox served as the reference antioxidant. The calibration curve included eight concentrations ranging from 5 to 200 μM, with dilution ratios of 1:2 and 1:5, and three replicates for each. All sample measurements were performed in triplicate, and results were reported in μmol TE/g DW. IC₅₀ values were also calculated, indicating the concentration of extract needed to inhibit 50% of the initial ABTS radical activity. Concentrations used to estimate IC₅₀ ranged from 0.01 to 10 mg/mL.

#### 2.12.3 FRAP assay

The FRAP (Ferric Reducing Antioxidant Power) assay was employed to evaluate the ferric ion-reducing ability of the sample. According to the standard procedure, a 10 mM TPTZ (2,4,6-tris(2-pyridyl)-*s*-triazine) solution was prepared by dissolving 33.5 mg of TPTZ in 10 mL of 40 mM hydrochloric acid. To prepare the FRAP working solution, 1 mL of this TPTZ solution was combined with 1 mL of a 20 mM ferric chloride solution and 10 mL of 0.3 M acetate buffer adjusted to pH 3.6. The mixture was then preheated to 37 °C. Following this, 0.15 mL of the hydroalcoholic extract was added to 2.85 mL of the FRAP working solution, and the samples were incubated in darkness for 4 min at 37 °C. After incubation, absorbance was measured at 593 nm using a V-730 UV–Visible/NIR spectrophotometer operated *via* the Spectra Manager™ Suite software (Jasco Inc., Easton, MD, USA). The absorbance values of blank FRAP solutions, which did not contain any sample, were recorded and subtracted from those of the test solutions. Trolox was used as the reference antioxidant, and results were expressed in μmol Trolox equivalents per gram of dry weight (μmol TE/g DW). A calibration curve was created using eight Trolox concentrations ranging from 5 to 200 μM, prepared with dilution ratios of 1:2 and 1:5, and each concentration was tested in triplicate.

### 2.13 Hydrogen sulfide measurement in a cell-free assay

The ability of OME to release H_2_S was assessed using a fluorescence-based assay with the fluorescent probe sulfidefluor-7-acetoxymethyl ester (SF7-AM, 10 μM). The samples were dissolved in distilled water at concentrations ranging from 1 to 1000 μg/mL. SF7-AM was added to each sample, and the mixture was incubated in a black 96-well plate in the dark at 37 °C. The concentration of H_2_S released was determined through a calibration curve of Na_2_S (50 nM–200 μM). The fluorescence was measured at 15 min of incubation with the samples (ex: 475 nm; em: 500–550 nm). Results were expressed as mean ± SEM (*n* = 3) and reported as μM.

### 2.14 Cell culture and experiments

Bovine aortic endothelial cells (BAECs) were purchased from iXCells Biotechnologies (Cat# 10BO-001) and were cultured in Modified Dulbecco's Medium (DMEM) supplemented with 2 mmol/L glutamine, 10% heat-inactivated fetal bovine serum, and 50 U/mL penicillin/streptomycin. Cells were maintained at 37 °C in a humidified incubator with 5% CO₂ and 95% air. The culture medium was replaced daily until the cells reached confluence. Passages 6–10 were used in all experiments. The beneficial effect of OME was assessed by an *in vitro* model of lipid-overload in BAEC induced by exposure to sodium palmitate (NaP; 100 μM) for 4 h (Smimmo et al., 2024). The results were compared with a reference compound, the H₂S donor sodium sulfide (Na₂S; 100 μM). In detail, after serum deprivation for 24 h, cells were pre-incubated with OME (100 μg/mL) or vehicle, 30 min before the addition of NaP for 4 h.

### 2.15 MTT assay

BAEC were seeded in 96-well plates at a density of 5 × 10^4^ cells per well and incubated at 37 °C. After 24 h, cells were exposed to OME (1, 10, 100, and 1000 μg/mL) for 4 h and 30 min. Subsequently, MTT solution (5 mg/mL in 1× PBS; Merck, Italy) was added to each well and plates were incubated for an additional 3 h at 37 °C. The medium was then removed, and dimethyl sulfoxide (DMSO) was added to solubilize the formazan crystals and stirred for 15 min. Absorbance was recorded at 570 nm using a microplate reader (Thermo Scientific Multiskan GO). Results were expressed as mean ± SEM (*n* = 4) and reported as % of cell viability relative to vehicle mean (set at 100%).

### 2.16 Hydrogen sulfide measurement in BAECs

BAECs were first incubated in phenol red-free DMEM for 30 min at 37 °C. Thereafter, BAEC were incubated in DMEM without phenol red for 30 min at 37 °C. After incubation, the medium was replaced with a medium containing SF7-AM (10 μM) for 30 min at 37 °C (Casertano et al., 2023). Cells were then treated with different concentrations of OME (10, 100, and 1000 μg/mL), glucoraphasatin (1, 10, 100, and 1000 μg/mL) or vehicle to measure live H_2_S production. The fluorescence was recorded every minute for the first 5 min and then at 15-min intervals for up to 2 h. Results were expressed as mean ± SEM (*n* = 3) and reported as fluorescence intensity.

### 2.17 NOx levels

Nox levels, expressed as the sum of nitrate (NO_3_^−^) and nitrite (NO_2_^−^), were measured in BAEC treated with OME (100 μg/ml) 30 min before NaP challenge (100 μM) for 4 h. In detail, cells were plated in 24-well culture plates at a density of 5 × 10^4^ cells/ well and allowed to adhere for 24 h. After incubation, supernatants were collected and incubated in a 96-well plate with cadmium (50 mg/well) for 1 h to convert the inorganic anion NO_3_^−^ to NO_2_^−^ (Olivencia et al., 2023). After centrifugation at 8000 *g*, total NOx content was determined using a fluorometric method by Promega Glomax explorer and calculated through a standard curve of sodium nitrite (NaNO_2_, 50–2000 nM). Results were expressed as mean ± SEM (*n* = 4) and reported as nM.

### 2.18 Intracellular reactive oxygen species measurement

Reactive oxygen species (ROS) were measured in BAEC treated with OME (100 μg/ml) 30 min before NaP treatment (100 μM) for 4 h. ROS generation was assessed using the fluorescent probe 2′,7′-dichlorofluorescein (DCF, Sigma-Aldrich, Milan, Italy) (Esposito et al., 2024). BAEC were seeded in black 96-well plates (Corning, USA) at a density of 5 × 10^4^ cells/well and, upon reaching ∼80% confluence. After washing with PBS, cells were incubated for 30 min with 10 μM DCF in HBSS containing 1% FBS. Fluorescence intensity was measured using a microplate reader (ex: 485 nm; em: 538 nm; GloMax®-Multi Detection System, Promega). Results were expressed as mean ± SEM (n = 4) and expressed as relative DCF fluorescence normalized to the vehicle.

### 2.19 Statistics

Unless otherwise stated, all the experimental results were expressed as the mean ± standard deviation (SD) of 3 repetitions or standard error (SEM). Graphics were plotted using Microsoft® Excel 2013 and GraphPad Prism 8 softwares. The statistical analyses were carried out using the SPSS 27.0 software (IBM Corporation, NY, USA) by using one-way ANOVA followed by Tukey's post-hoc test. Significant differences were accepted at the 95% confidence level or with a *p* value <0.05.

## 3 Results and discussion

### 3.1 Validation of GSLs quantitative HPLC-qQq-MS/MS analysis

In order to identify the microgreen sample with the highest GSLs content, a specific HPLC-qQq-MS/MS method for the quantitative GSLs analysis was thoroughly validated in terms of linearity, sensitivity, accuracy, precision, reproducibility, and carry-over. Method sensitivity was determined by calculating the LOD and LOQ values for the selected glucosinolates, namely glucoraphanin, glucoraphenin, and glucoraphasatin (Supplementary Material, Table 2). These compounds were chosen for method validation as they represent the most abundant and representative GSLs in radish microgreens (Demir et al., 2023; Jauregui et al., 2025). The three compounds exhibited a great sensitivity of detection, with LOD values of 0.68 ± 0.40 ppb, 0.53 ± 0.19 ppb, and 0.25 ± 0.01 ppb for glucoraphanin, glucoraphenin, and glucoraphasatin, respectively. Moreover, the compounds displayed notable sensitivity for the quantification, with LOQ values of 2.06 ± 1.20 ppb, 1.61 ± 0.59 ppb, and 0.76 ± 0.03 ppb for glucoraphanin, glucoraphenin, and glucoraphasatin, respectively. Given that these limits are significantly lower than the concentrations measured in the analyzed samples, the method can be considered highly sensitive and suitable for both detection and quantification of GSLs. Analytical reproducibility was evaluated through repeated injections of QC samples, and all compounds exhibited RSD% values <5%, highlighting high reproducibility both in terms of peak area and retention time and supporting the reliability of the method. In addition, carry-over tests confirmed a negligible residual signal (<0.1%) for the three GSLs. Precision and accuracy were evaluated by performing intra-day and inter-day analyses on a standard mixture of GSLs at three concentration levels. As reported in Supplementary Material Table 3, intra-day and inter-day precision ranged from 0.42% to 8.80% and from 1.72% to 8.85%, respectively. Accuracy ranged from −0.50% to 1.35% for intra-day assessments and from −8.61% to 0.79% for inter-day measurements. Notably, the direct quantification of intact GSLs represents a significant methodological advantage compared to conventional approaches based on desulfo-GSLs, as it avoids additional enzymatic steps and enables a more accurate reflection of the native GSLs profile. For these reasons, in the present study we deliberately selected the direct quantification of intact GSLs in order to obtain a more accurate characterization of the native glucosinolate composition of radish microgreens. Overall, these analytical parameters confirm that the HPLC-qQq-MS/MS method ensures robust reproducibility and reliability for the quantification of the three GSL standards. To ensure accurate quantification of intact GSLs, preliminary experiments were conducted to evaluate the effect of extraction temperature on endogenous myrosinase activity. Specifically, glucoraphasatin was incubated with myrosinase at different temperatures, and the extent of substrate hydrolysis was monitored over time (Supplementary Material, Fig. S1). These experiments demonstrated that increasing temperatures markedly reduced enzymatic activity, with near-complete suppression observed under the selected extraction conditions. Based on these findings, the extraction temperature of 75 °C was selected as a suitable compromise to ensure effective inactivation of endogenous myrosinase while preserving GSL structural integrity. This choice is consistent with previous literature reporting efficient myrosinase inactivation at elevated temperatures (*e.g.*, 80 °C) without significant GSLs degradation (Piccolo, Maisto, Marino Cerrato, et al., 2024).

### 3.2 Quantitative determination of GSLs by HPLC-qQq-MS/MS in microgreens samples

In order to maximize the GSLs production, radish microgreens were cultivated under different light intensities and harvested at multiple time points. Specifically, two light settings were used, red light and blue light, and microgreens were harvested at 3, 6, and 9 days after sowing (T1, T2, and T3, respectively). Quantitative analysis revealed significant differences in GSLs accumulation depending on both the light condition and the harvesting time (Table 1). Glucoraphasatin and glucoraphenin were the predominant GSLs detected in all analyzed samples, while glucoraphanin was detected only after 3 harvesting days. Under warm RL, total GSL content peaked at 6 days (T2), reaching 70.71 mg/g DW, significantly higher than at T1 and T3 (*p* < 0.005). Glucoraphasatin increased from 52.24 ± 0.38 mg/g DW at T1 to 58.98 ± 1.65 mg/g DW at T2, then was reduced to 43.59 ± 1.02 mg/g DW at T3. A similar declining trend was observed for glucoraphenin, which passed from 15.58 ± 0.43 mg/g DW at T1 to 8.11 ± 0.45 mg/g DW at T3. Under BL conditions, total GSL levels were consistently lower, with a maximum of 49.61 mg/g DW observed, also at T2. Glucoraphasatin remained relatively stable across time points (38.24–39.22 mg/g DW), while glucoraphenin decreased from 7.26 ± 0.14 mg/g DW at T1 to 6.21 ± 0.31 mg/g DW at T3. Overall, the results suggest that cultivating radish microgreens under RL conditions and harvesting after 6 days is the most effective strategy to promote the accumulation of GSLs and consequently increase their nutraceutical potential. These results are in line with data from other authors who reported that the GSLs concentration in radish microgreens can change depending on the light conditions and the time of cultivation (Michalczyk, 2025). Specifically; Demir and his colleagues also found that the most representative GSL of microgreens is glucoraphasatin; and the maximum concentrations approximately of 23–24 mg/g DW of which were achieved under red LED irradiation (Demir et al., 2023). The substantially higher GSL levels observed in the present study may be attributed to several factors. First; glucosinolate accumulation is known to be strongly influenced by genetic background; and differences in cultivar can significantly affect both total content and individual GSL profiles. In addition; variations in light conditions; including spectral composition; intensity; and light combinations; can markedly modulate glucosinolate biosynthesis; as previously reported for *Brassicaceae* (Zhou et al., 2023). Furthermore; methodological differences may also contribute to the observed discrepancy. It is worth noting that the higher GSL levels observed in this study may also reflect the use of an analytical approach based on intact GSL quantification; which avoids potential underestimation associated with desulfation-based methods commonly reported in literature (Gallaher et al., 2012). Our data are consistent with previous evidence showing that GSL accumulation in *Brassicaceae* microgreens is strongly influenced by both developmental stage and light quality. The marked peak observed at six days (T2) under RL (70.71 mg of total GSLs content/g DW) reflects a physiological window in which primary and secondary metabolism are optimally balanced; leading to the active biosynthesis of defense-related compounds (Michalczyk, 2025).

**Table 1:** HPLC-qQq-MS/MS GSLs quantitative analysis of radish microgreens.

Compound (mg/ g DW ± SD)	RL			BL		
	T1	T2	T3	T1	T2	T3
Glucoraphasatin	52.24 ± 0.38^b^	58.98 ± 1.65^a^	43.59 ± 1.02^c^	38.24 ± 0.38^d^	39.98 ± 0.69^d^	39.22 ± 0.23^d^
Glucoraphenin	15.58 ± 0.43^a^	11.73 ± 0.97^b^	8.11 ± 0.45^c^	7.26 ± 0.14^c,d^	9.63 ± 0.21^c^	6.21 ± 0.31^d^
Glucoraphanin	0.05 ± 0.01^a^	< LOQ	< LOQ	0.04 ± 0.01^a^	< LOQ	< LOQ
Total GSLs	67.87	70.71	51.70	45.54	49.61	45.43

Data are expressed as mean value (mg component/g microgreens DW) ± SD of three repetitions (*n* = 3). Total GSLs content was calculated as sum of glucoraphasatin, glucoraphanin, and glucoraphanin contents for each experimental condition. Abbreviations: T1, 3 days; T2, 6 days; T3, 9 days; RL, red light; BL, blue light; ND, not detected. For each GSLs, mean values with different superscript letters (a, b, c, d) are significantly different by Tukey's multiple comparison test, calculated with *p* < 0.05, calculated between different harvesting times (T1, T2, T3) and light settings (RL, BL) within the same row.

The subsequent reduction of GSL concentration observed during microgreen development (T3) may be a consequence of selective glucosinolate metabolism, as well as dilution of their concentration during tissue expansion (Bellostas et al., 2007). Irradiation conditions also exerted a decisive role in microgreens development and GSL accumulation. Exposure to warm light; which is relatively richer in red wavelengths; led to a significantly higher content of glucosinolates compared to cold light. This effect can be explained by the ability of red spectra to stimulate photoreceptor-mediated signaling cascades. In particular; red light enhances the activity of the transcription factor elongated hypocotyl 5 (Hy5); which regulates the expression of biosynthetic genes associated with aliphatic glucosinolates. Huseby et al. (2013) demonstrated that Hy5 acts downstream of light perception and controls the expression of several transcription factors of the myeloblastosis family; including myeloblastosis 28 (Myb28); myeloblastosis 29 (Myb29); and myeloblastosis 76 (Myb76); which are key regulators of the aliphatic glucosinolate biosynthetic pathway (Huseby et al., 2013). More recently; Wang et al. confirmed that red light strengthens this Hy5-dependent transcriptional network; resulting in the upregulation of glucosinolate biosynthetic genes and the subsequent accumulation of aliphatic glucosinolates (Wang et al., 2021).

### 3.3 Qualitative profile of GSLs by PIS approach in OM samples

After identifying the microgreen sample exhibiting the highest glucosinolate concentration (OM, optimized microgreen), a qualitative profiling of the less abundant GSL species was subsequently carried out to provide a comprehensive assessment of its chemical composition. The qualitative identification of GSLs in microgreen samples was carried out using the PIS approach, which has proven to be a highly effective strategy for simplifying the profiling of complex natural matrices. The method was initially developed by screening 14 analytical standards representing a wide structural diversity, particularly in terms of side-chain variations, thus enabling the construction of a generic and robust recognition tool for diverse GSLs. The PIS method involved monitoring the diagnostic fragment ions at *m/z* 259, 97, and 96, which correspond to characteristic sulfate and thioglucose moieties released during tandem mass spectrometry. By applying this approach across a broad *m/z* window (100−1000), the technique allowed for the sensitive and selective detection of precursor ions corresponding to intact GSLs. This methodology enabled the reliable identification of several known GSLs in microgreen extracts, including glucoraphanin (*m/z* 436.3), glucoraphenin (*m/z* 434.4), glucoraphasatin (*m/z* 418.3), glucobrassicin (*m/z* 447.4), and 4-methoxyglucobrassicin (*m/z* 477.4). Their identities were confirmed by co-elution with analytical standards and comparison of MS/MS fragmentation patterns and retention times. In addition to the GSLs confirmed by authentic standards, the method allowed the detection of several additional precursor ions with characteristic GSL-derived fragmentations. These compounds exhibited clear MS/MS profiles consistent with the core structure of glucosinolates, suggesting that they may belong to structurally related analogues. The results of the qualitative analysis are reported in Table 2. They include both glucosinolates previously reported in literature (7-methylthioheptyl GSL, *m/z* 462.3; sinapoylglucoraphenin, *m/z* 640.5; benzoylglucoraphenin, *m/z* 538.4) and three unknown compounds. The PIS strategy was essential to selectively track compounds with display diagnostic fragments of the GSL core in MS/MS experiments. However, the ion with *m/z* 259, which is peculiar of GSLs fragmentation, was not detected in all compounds. For instance, sinapoylglucoraphenin is characterized by only sulfate-related fragments with *m/z* 96 and 97. Its fragmentation pattern displayed the diagnostic high-mass ions at *m/z* 497, 481, and 465, which are consistent with fragments derived by the substituted glucose moiety. Similarly, the unknown compounds with *m/z* 539.6 and 607.7 were putatively identified as GSLs due to the presence of the highly diagnostic sulfate ions at *m/z* 97 and 96. The absence of the fragment ion with *m/z* 259 suggested a chemical modification of the glucose moiety. In addition, these unknown compounds displayed a consistent neutral loss of 15 Da from the parent ion, indicative of a methyl group elimination which is typically associated with the methylsulfinylbutenyl moiety, previously reported in glucoraphenin. Taken together, these findings suggest that the unknown metabolites are glucoraphenin-derived GSLs bearing structural modifications at the glucose moiety. The presence of unknown features with consistent GSL-like fragmentation patterns may reflect natural structural variants arising from side-chain modifications such as esterification of the sugar moiety, highlighting the biochemical diversity of GSLs in *Brassicaceae* microgreens. Although the proposed structures of unknown compounds are supported by consistent MS/MS fragmentation patterns (*e.g.*, characteristic neutral losses and diagnostic sulfate ions), these compounds should be considered as putatively identified glucosinolate-related metabolites. Further structural confirmation by orthogonal techniques (*e.g.*, NMR or comparison with reference standards) would be required for definitive identification. Moreover, the high selectivity and sensitivity of the PIS approach make it a powerful tool for the detection of low-abundance metabolites, enhancing the capacity to explore the chemical complexity of glucosinolate profiles in OM sample.

**Table 2:** Tentative identification of GSLs in OM sample using PIS mode.

Compound	Rt	*m/z*	Fragmentation
Glucoraphanin	2.90	436.3	371.9 [M-H-CH_4_OS]^−^, 258.8 [M-H-C_6_H_11_NOS_2_]^−^, 96.9 [M-H-C_12_H_22_NO_6_S_2_]^−^, 96.0 [M-H- C_12_H_23_NO_6_S_2_]^−^
Glucoraphenin	3.10	434.4	418.8 [M-H-CH_3_]^−^, 274.9 [M-H-C_6_H_9_NO_2_S]^−^, 258.9 [M-H-C_6_H_9_NOS_2_]^−^, 240.8 [M-H-C_6_H_11_NO_2_S_2_]^−^, 194.8 [M-H-C_6_H_9_NO_4_S_2_]^−^, 96.9 [M-H-C_12_H_19_NO_6_S_2_]^−^, 96.0 [M-H-C_12_H_20_NO_6_S_2_]^−^
7-Methylthioheptyl GSL	5.40	462.3	275.0 [M-H-C_9_H_17_NOS]^−^, 259.0 [M-H-C_9_H_17_NS_2_]^−^, 240.9 [M-H-C_9_H_19_NOS_2_]^−^, 96.9 [M-H-C_15_H_27_NO_5_S_2_]^−^, 96.0 [M-H-C_15_H_28_NO_5_S_2_]^−^
Glucoraphasatin	7.20	418.3	274.9 [M-H-C_6_H_9_NOS]^−^, 258.9 [M-H-C_6_H_9_NS_2_]^−^, 240.8 [M-H-C_6_H_11_NOS_2_]^−^, 194.8 [M-H-C_6_H_9_NO_4_S_2_]^−^, 96.9 [M-H-C_12_H_19_NO_5_S_2_]^−^, 96.0 [M-H-C_12_H_20_NO_5_S_2_]^−^
Glucobrassicin	7.50	447.4	366.9 [M-H-SO_3_]^−^, 290.6 [M-H-C_10_H_8_N_2_]^−^, 258.8 [M-H-C_10_H_8_N_2_S]^−^, 240.6 [M-H-C_10_H_10_N_2_O_2_S]^−^, 204.7 [M-H-C_6_H_10_O_8_S]^−^, 96.9 [M-H-C_16_H_18_N_2_O_5_S]^−^, 96.0 [M-H-C_16_H_19_N_2_O_5_S]^−^
Unknown GSL	7.90	539.6	474.8, 437.2, 411.2, 375.2, 317.2, 290.8, 240.9, 191.9, 169.2, 121.8, 96.9, 96.0
4-Methoxyglucobrassicin	9.35	477.4	290.7 [M-H-C_11_H_10_N_2_O]^−^, 274.9 [M-H-C_11_H_10_N_2_O_2_]^−^, 258.8 [M-H-C_11_H_10_N_2_OS]^−^, 194.8 [M-H-C_11_H_10_N_2_O_4_S]^−^, 96.9 [M-H-C_17_H_20_N_2_O_6_S]^−^, 96.0 [M-H-C_17_H_21_N_2_O_6_S]^−^
Unknown GSL	9.50	607.7	592.1, 543.1, 464.0, 448.0, 432.1, 292.0, 190.1, 96.9, 96.0
Unknown GSL	9.95	607.8	592.2, 543.1, 464.1, 448.1, 432.1, 292.1, 190.1, 97.0, 96.0
Sinapoylglucoraphenin	10.25	640.5	625.1 [M-H-CH_3_]^−^, 497.0 [M-H-C_6_H_9_NOS]^−^, 481.0 [M-H-C_6_H_9_NO_2_S]^−^, 465.1 [M-H-C_6_H_9_NOS_2_]^−^, 96.9 [M-H-C_23_H_29_NO_10_S_2_]^−^, 96.0 [M-H-C_23_H_30_NO_10_S_2_]^−^
Benzoylglucoraphenin	11.10	538.4	522.9 [M-H-CH_3_]^−^, 394.7 [M-H-C_6_H_9_NOS]^−^, 259.0 [M-H-C_13_H_13_NO_2_S_2_]^−^, 240.9 [M-H-C_13_H_15_NO_3_S_2_]^−^, 96.9 [M-H-C_19_H_23_NO_7_S_2_]^−^, 96.0 [M-H-C_19_H_24_NO_7_S_2_]^−^

The assignments are based on *m/z* values, retention times, and diagnostic fragment ions observed in MS/MS acquisition mode.

### 3.4 Qualitative profile of polyphenols by HPLC-qQq-MS/MS in OM samples

In order to comprehensively evaluate the nutraceutical potential of OM, a qualitative characterization of its polyphenolic profile was performed, complementing the GSL assessment. The qualitative profile of polyphenols in OM sample was carried out using HPLC-HESI-MS/MS analysis in positive and negative acquisition modes. The retention time, quasi-molecular ion, and the fragment ions were listed in Table 3. Based on the comparison with literature data, 54 compounds were putatively identified. The identity of 3 compounds (rutin, ferulic acid, and sinapic acid) was confirmed by comparison with analytical standards. Anthocyanins are among the most abundant phenolic compounds in OM sample. The HPLC-HESI-MS/MS analysis in positive acquisition mode displayed the content of cyanidin-based structures, occurring mainly in glycosylated and acylated forms. The acylation profile was particularly enriched in sinapoyl residues, a common moiety of radish phenylpropanoid metabolism, which is known to produce high levels of sinapic acid. The anthocyanin profile revealed a wide structural variety, including mono-, di-, and triglycosylated derivatives, and further modified by malonyl, coumaroyl, feruloyl, and especially sinapoyl groups. Compound identification was supported by the presence of diagnostic fragment ions resulting from sequential losses of sugar and acyl moieties. In particular, the loss of 162 Da indicated cleavage of a glucose unit, 146 Da for rhamnose, and 86 Da for malonic acid. Additionally, characteristic neutral losses corresponding to *p*-coumaric acid (146 Da), ferulic acid (176 Da), and sinapic acid (206 Da) were consistently observed across multiple compounds, confirming their acylation pattern. Polyphenols are widely distributed in OM sample and contribute significantly to its antioxidant profile. The HPLC-HESI-MS/MS analysis in negative ionization mode allowed the detection of a broad array of phenolic structures, including phenolic acids, flavonols, and flavan-3-ols, predominantly present in glycosylated and acylated forms. The profile was particularly enriched in hydroxycinnamic acid derivatives, such as sinapic, ferulic, and *p*-coumaric acid esters, often conjugated to quinic acid or hexose moieties. Kaempferol and quercetin glycosides were also abundant, occurring as mono- and diglycosylated forms and as derivatives acylated with phenolic acids. Compound identification was supported by diagnostic MS/MS fragmentation patterns characteristic of phenolic compounds. The loss of 162 Da indicated the cleavage of a glucose unit, while 132 Da was attributed to pentose losses. Acylations were confirmed by neutral losses corresponding to malonic acid (86 Da), *p*-coumaric acid (146 Da), ferulic acid (176 Da), and sinapic acid (206 Da). The marked presence of sinapoylated derivatives underscores the biochemical specificity of OM sample for sinapate-derived polyphenols biosynthesis, further supporting the nutritional interest of this microgreen matrix.

**Table 3:** Polyphenols and anthocyanins identified or putatively identified in OME sample by their MS spectra according to the literature by HPLC-MS analysis in positive and negative acquisition modes.

Compound	Adduct	Rt	*m/z*	Fragmentation
Methyl citric acid	[M-H]^−^	1.51	190.9	172.8 [M-H-H_2_O]^−^; 146.9 [M-H-CO_2_]^−^; 102.8 [M-H-2CO_2_]^−^
Salicyloyl-*O*-Hexoside	[M-H]^−^	3.33	299.1	239.1 [M-H-C_2_H_2_O_2_]^−^; 209.2 [M-H-C_3_H_6_O_3_]^−^; 179.2 [M-H-C_4_H_8_O_4_]^−^; 137.0 [M-H-Hex]^−^
Feruloyl-*O*-Hexoside	[M-H]^−^	6.70	355.2	295.2 [M-H-C_2_H_2_O_2_]^−^; 265.3 [M-H-C_3_H_6_O_3_]^−^; 234.9 [M-H-C_4_H_8_O_4_]^−^; 217.1 [M-H-C_4_H_10_O_5_]^−^; 193.0 [M-H-Hex]^−^; 175.01 [M-H-Hex-H_2_O]^−^
Sinapoyl-gentiobiose	[M-H]^−^	6.97	547.2	367.1 [M-H-Hex-H_2_O]^−^; 341.2 [M-H-Sin]^−^; 223.1 [M-H-2Hex]^−^; 205.0 [M-H-2Hex-H_2_O]^−^; 190.0 [M-H-2Hex-H_2_O-CH_3_]^−^; 175.1 [M-H-2Hex-H_2_O-2CH_3_]^−^
Sinapoyl-glucoside	[M-H]^−^	7.20	385.2	367.2 [M-H-H_2_O]^−^; 325.2 [M-H-C_2_H_2_O_2_]^−^; 295.2 2 [M-H-C_3_H_6_O_3_]^−^; 265.1 [M-H-C_4_H_8_O_4_]^−^; 247.0 [M-H-C_4_H_10_O_5_]^−^; 223.1 [M-H-Hex]^−^; 205.0 [M-H-Hex-H_2_O]^−^
Cyanidin 3-*O*-(sinapoyl)-diglucoside-5-*O*-glucoside	[M]^+^	7.38	979.3	817.4 [M-Hex]^+^; 449.3 [M-2Hex-Sin]^+^; 287.0 [M-3Hex-Sin]^+^;
Cyanidin 3-*O*-(feruloyl)sophoroside-5-*O*-glucoside isomer 1	[M]^+^	7.40	949.4	787.3 [M-Hex]^+^; 449.3 [M-2Hex-Fer]^+^; 287.0 [M-3Hex-Fer]^+^
Delphinidin 3-*O*-rutinoside-5-*O*-glucoside	[M]^+^	7.89	773.2	729.4 [M-CO_2_]^+^; 611.2 [M-Hex]^+^; 465.4 [M-Hex-Rha]^+^; 449.0 [M-2Hex]^+^; 303.0 [M-2Hex-Rha]^+^
Kaempferol-3-*O*-sophotrioside-7-*O*-glucoside	[M-H]^−^	7.92	933.3	771.3 [M-H-Hex]^−^; 609.2 [M-H-2Hex]^−^
Kaempferol 3-*O*-sophoroside-7-*O*-glucoside	[M-H]^−^	8.09	771.2	609.2 [M-H-Hex]^−^; 447.2 [M-H-2Hex]^−^; 285.1 [M-H-3Hex]^−^
Quercetin 3-*O*-Caffeoylsophoroside-7-*O*-glucoside	[M-H]^−^	8.42	949.3	787.1 [M-H-Hex]^−^; 625.1 [M-H-2Hex]^−^; 463.1 [M-H-3Hex]^−^; 301.0 [M-H-Caf-3Hex]^−^
Quercetin 3-*O*-glucoside-7-*O*-glucoside	[M-H]^−^	8.69	625.2	463.1 [M-H-Hex]^−^; 301.2 [M-H-2Hex]^−^
Quercetin 3-*O*-rutinoside-7-*O*-glucoside	[M-H]^−^	8.74	771.2	609.2 [M-H-Hex]^−^; 463.2 [M-H-Hex-Rha]^−^; 301.0 [M-H-2Hex-Rha]^−^
Rutin⁎	[M-H]^−^	8.91	609.2	463.2 [M-H-Rha]^−^; 447.1 [M-H-Hex]^−^; 301.1 [M-H-Hex-Rha]^−^; 283.0 [M-H-Hex-Rha-H_2_O]^−^
Kaempferol 3-*O*-caffeoyldiglucoside-7-*O*-glucoside	[M-H]^−^	9.09	965.2	947.3 [M-H-H_2_O]^−^; 803.2 [M-H-Hex]^−^; 785.3 [M-H-Hex-H_2_O]^−^; 285.0 [M-H-3Hex-Caf]^−^
Kaempferol 3-*O*-caffeoylsophorotrioside-7-*O*-glucoside	[M-H]^−^	9.17	1095.2	1050.9 [M-H-CO_2_]^−^; 933.2 [M-H-Hex]^−^; 771.2 [M-H-2Hex]^−^
Cyanidin 3-*O*-(glucopyranosyl-sinapoyl)diglucoside-5-*O*-glucoside	[M]^+^	9.33	1141.3	1123.3 [M-H_2_O]^+^; 978.8 [M-Hex]^+^; 817.1 [M-2Hex]^+^; 449.2 [M-3Hex-Sin]^+^
Cyanidin 3-*O*-(coumaroyl)sophoroside-5-*O*-glucoside	[M]^+^	9.60	919.3	901.5 [M-H_2_O]^+^; 757.3 [M-Hex]^+^; 735.4 [M-Hex-H_2_O]^+^; 595.0 [M-2Hex]^+^; 449.0 [M-2Hex-Cou]^+^; 287.2 [M-3Hex-Cou]^+^
Ferulic acid⁎	[M-H]^−^	9.63	193.0	178.0 [M-H-CH_3_]^−^; 149.0 [M-H-CO_2_]^−^; 133.9 [M-H-CO_2_-CH_3_]^−^
Cyanidin 3-*O*-(feruloyl)sophoroside-5-*O*-glucoside isomer 2	[M]^+^	9.75	949.3	787.3 [M-Hex]^+^; 449.3 [M-2Hex-Fer]^+^; 287.0 [M-3Hex-Fer]^+^
Sinapic acid⁎	[M-H]^−^	9.91	223.2	208.0 [M-H-CH_3_]^−^; 179.0 [M-H-CO_2_]^−^; 164.0 [M-H-CH_3_-CO_2_]^−^; 149.0 [M-H-2CH_3_-CO_2_]^−^;
Cyanidin 3-*O*-(feruloyl)diglucoside-5-*O*-(malonyl)glucoside isomer 1	[M]^+^	9.92	1035.4	991.5 [M-CO_2_]^+^; 949.4 [M-Mal]^+^; 787.3 [M-Hex-Mal]^+^; 625.2 [M-2Hex-Mal]^+^; 535.3 [M-2Hex-Fer]^+^; 449.3 [M-2Hex-Fer-Mal]^+^; 287.1 [M-3Hex-Fer-Mal]^+^
Quercetin 3-*O*-sophoroside-7-*O*-sinapoylrhamnoside	[M-H]^−^	9.96	977.3	815.1 [M-H-Hex]^−^; 771.3 [M-H-Sin]^−^; 609.3 [M-H-Hex-Sin]^−^; [M-H-Hex-Rha-Sin]^−^; 301.0 [M-H-2Hex-Rha-Sin]^−^
Cyanidin 3-*O*-(glucosyl)(sinapoyl)(coumaroyl)sophoroside-5-*O*-glucoside	[M]^+^	10.07	1287.1	1125.3 [M-Hex]^+^; 963.5 [M-2Hex]^+^; 611.2 [M-2Hex-Sin-Cou]^+^
Cyanidin 3-*O*-(glucosyl)(sinapoyl)(feruloyl)sophoroside-5-*O*-glucoside isomer 1	[M]^+^	10.20	1317.2	1155.3 [M-Hex]^+^; 993.4 [M-2Hex]^+^; 787.4 [M-2Hex-Sin]^+^; 611.4 [M-2Hex-Sin-Fer]^+^
Cyanidin 3-*O*-(coumaroyl)sophoroside-5-*O*-(malonyl)glucoside	[M]^+^	10.32	1005.4	757.3 [M-Hex-Mal]^+^; 697.0 [M-Hex-Cou]^+^; 595.2 [M-2Hex-Mal]^+^; 535.3 [M-2Hex-Cou]^+^; 287.1 [M-3Hex-Cou-Mal]^+^
Cyanidin 3-*O*-(feruloyl)(sinapoyl)sophoroside-5-*O*-glucoside	[M]^+^	10.36	1155.4	1111.0 [M-CO_2_]^+^; 993.2 [M-Hex]^+^; 817.4 [M-Hex-Fer]^+^; 787.1 [M-Hex-Sin]^+^; 449.2 [M-2Hex-Sin-Fer]^+^
Cyanidin 3-*O*-diferuloylsophoroside-5-*O*-glucoside isomer 1	[M]^+^	10.44	1125.3	963.1 [M-Hex]^+^; 449.1 [M-2Hex-2Fer]^+^; 431.2 [M-2Hex-2Fer-H_2_O]^+^
Cyanidin 3-*O*-(coumaroyl)(sinapoyl)diglucoside-5-*O*-(malonyl)glucoside	[M]^+^	10.46	1227.3	1183.4 [M-CO_2_]^+^; 1141.3 [M-Mal]^+^; 1021.1 [M-Sin]^+^; 979.3 [M-Hex-Mal]^+^; 697.5 [M-2Hex-Sin]^+^; 535.2 [M-3Hex-Sin]^+^; 491.1 [M-3Hex-Sin-CO_2_]^+^; 449.2 [M-3Hex-Sin-Mal]^+^
Quercetin 3-*O*-sinapoyltriglucoside-7-*O*-glucoside	[M-H]^−^	10.48	1155.3	993.1 [M-H-Hex]^−^; 949.1 [M-H-Sin]^−^; 931.4 [M-H-Sin-H_2_O]^−^; 787.3 [M-H-Hex-Sin]^−^; 769.3 [M-H-Hex-Sin-H_2_O]^−^
Cyanidin 3-*O*-(glucosyl)(sinapoyl)(feruloyl)sophoroside-5-*O*-glucoside isomer 2	[M]^+^	10.49	1317.3	1155.5 [M-Hex]^+^; 993.3 [M-2Hex]^+^; 611.4 [M-2Hex-Sin-Fer]^+^
Cyanidin-3-*O*-(sinapoyl)sophoroside-5-*O*-(malonyl)glucoside	[M]^+^	10.50	1065.3	1021.2 [M-CO_2_]^+^; 979.3 [M-Mal]^+^; 903.5 [M-Hex]^+^; 817.4 [M-Hex-Mal]^+^; 697.3 [M-Hex-Sin]^+^; 655.3 [M-2Hex-Mal]^+^; 535.2 [M-2Hex-Sin]^+^; 491.2 [M-2Hex-Sin-CO_2_]^+^; 449.1 [M-2Hex-Sin-Mal]^+^
Kaempferol 3-*O*-sophoroside-7-*O*-sinapoylrhamnoside	[M-H]^−^	10.50	961.3	815.3 [M-H-Rha]^−^; 799.3 [M-H-Hex]^−^; 755.3 [M-H-Sin]^−^; 653.3 [M-H-Hex-Rha]^−^; 609.2 [M-H-Rha-Sin]^−^; 447.2 [M-H-Hex-Rha-Sin]^−^; 300.0 [M-H-2Hex-Rha-Sin]^−^
Kaempferol 3-*O*-hydroxyferuloylsophoroside-7-*O*-glucoside	[M-H]^−^	10.51	963.2	919.0 [M-H-CO_2_]^−^; 801.4 [M-H-Hex]^−^; 609.2 [M-H-Hex-HFer]^−^; 285.2 [M-H-3Hex-HFer]^−^
Cyanidin 3-*O*-(feruloyl)diglucoside-5-*O*-(malonyl)glucoside isomer 2	[M]^+^	10.53	1035.3	991.4 [M-CO_2_]^+^; 949.5 [M-Mal]^+^; 787.3 [M-Hex-Mal]^+^; 625.2 [M-2Hex-Mal]^+^; 535.2 [M-2Hex-Fer]^+^; 449.2 [M-2Hex-Fer-Mal]^+^; 287.2 [M-3Hex-Fer-Mal]^+^
Quercetin 3-*O*-hydroxyferuloylsophorotrioside-7-*O*-glucoside	[M-H]^−^	10.55	1141.2	979.3 [M-H-Hex]^−^; 935.3 [M-H-Hex-CO_2_]^−^; 917.1 [M-H-Hex-CO_2_-H_2_O]^−^; 787.0 [M-H-Hex-HFer]^−^
Kaempferol 3-*O*-sinapoylferuloylsophoroside-7-*O*-glucoside	[M-H]^−^	10.67	1153.3	991.3 [M-H-Hex]^−^; 947.3 [M-H-Hex-CO_2_]^−^; 815.4 [M-H-Hex-Fer]^−^; 785.2 [M-H-Hex-Sin]^−^; 767.2 [M-H-Hex-Sin-H_2_O]^−^
Cyanidin 3-*O*-(sinapoyl)(coumaroyl)-triglucoside-5-*O*-(malonyl)glucoside isomer 1	[M]^+^	10.85	1373.3	1287.4 [M-Mal]^+^; 963.5 [M-2Hex-Mal]^+^; 697.1 [M-2Hex-Sin-Cou]^+^
Cyanidin 3-*O*-diferuloylsophoroside-5-*O*-glucoside isomer 2	[M]^+^	10.97	1125.3	963.3 [M-Hex]^+^; 625.4 [M-2Hex-Fer]^+^; 449.2 [M-2Hex-2Fer]^+^
Disinapoylgentibiose isomer 1	[M-H]^−^	11.00	753.2	547.3 [M-H-Sin]^−^; 529.2 [M-H-Sin- H_2_O]^−^; 385.1 [M-H-Hex-Sin]^−^; 367.2 [M-H-Hex-Sin-H_2_O]^−^
Cyanidin 3-*O*-(sinapoyl)(coumaroyl)-triglucoside-5-*O*-(malonyl)glucoside isomer 2	[M]^+^	11.10	1373.3	1329.4 [M-CO_2_]^+^; 1287.3 [M-Mal]^+^;]^+^; 963.3 [M-2Hex-Mal]^+^; 697.2 [M-2Hex-Sin-Cou]^+^; 653.2 [M-2Hex-Sin-Cou-CO_2_]^+^
Feruloyl-sinapoyl-gentibiose	[M-H]^−^	11.20	723.2	705.2 [M-H-H_2_O]^−^; 547.3 [M-H-Fer]^−^; 529.2 [M-H-Fer-H_2_O]^−^; 517.3 [M-H-Sin]^−^; 499.2 [M-H-Sin-H_2_O]^−^; 385.1 [M-H-Hex-Fer]^−^; 367.1 [M-H-Hex-Fer-H_2_O]^−^; 223.0 [M-H-2Hex-Fer]^−^; 205.1 [M-H-2Hex-Fer-H_2_O]^−^
Cyanidin 3-*O*-(coumaroyl)(sinapoyl)diglucoside-5-*O*-(malonyl)glucoside isomer 1	[M]^+^	11.29	1211.4	1167.3 [M-CO_2_]^+^; 1125.0 [M-Mal]^+^; 963.3 [M-Hex-Mal]^+^; 887.0 [M-2Hex]^+^; 535.2 [M-2Hex-Cou-Sin]^+^; 491.1 [M-2Hex-Cou-Sin-CO_2_]^+^
Disinapoylgentibiose isomer 2	[M-H]^−^	11.30	753.3	547.2 [M-H-Sin]^−^; 529.2 [M-H-Sin- H_2_O]^−^; 385.2 [M-H-Hex-Sin]^−^; 367.1 [M-H-Hex-Sin-H_2_O]^−^
Cyanidin 3-*O*-(sinapoyl)(coumaroyl)-triglucoside-5-*O*-(malonyl)glucoside isomer 3	[M]^+^	11.36	1373.3	1329.4 [M-CO_2_]^+^; 1287.1 [M-Mal]^+^;]^+^; 963.2 [M-2Hex-Mal]^+^; 697.3 [M-2Hex-Sin-Cou]^+^; 653.3 [M-2Hex-Sin-Cou-CO_2_]^+^
Cyanidin 3-*O*-(sinapoyl)(sinapoyl)diglucoside-5-*O*-(malonyl)glucoside	[M]^+^	11.38	1271.2	1227.3 [M-CO_2_]^+^; 1185.3 [M-Mal]^+^; 1109.4 [M-Hex]^+^; 1023.4 [M-Hex-Mal]^+^; 535.2 [M-2Hex-2Sin]^+^; 491.2 [M-2Hex-2SIn-CO_2_]^+^; 449.3 [M-2Hex-2Sin-Mal]^+^
Cyanidin 3-*O*-(feruloyl)(sinapoyl)diglucoside-5-*O*-(malonyl)glucoside	[M]^+^	11.41	1241.3	1197.2 [M-CO_2_]^+^; 1155.3 [M-Mal]^+^; 1065.3 [M-Fer]^+^; 993.3 [M-Hex-Mal]^+^; 873.4 [M-Hex-Sin]^+^; 711.3 [M-2Hex-Sin]^+^; 535.3 [M-2Hex-Sin-Fer]^+^; 491.3 [M-2Hex-Sin-Fer-CO_2_]^+^; 449.3 [M-2Hex-Sin-Fer-Mal]^+^; 395.2 [M-2Hex-Sin-Fer-Mal-CO_2_]^+^
Quercetin 3-*O*-sinapoylsophoroside	[M-H]^−^	11.60	831.2	669.3 [M-H-Hex]^−^; 463.2 [M-H-Hex-Sin]^−^; 301.1 [M-H-2Hex-Sin]^−^
Cyanidin 3-*O*-(coumaroyl)(sinapoyl)diglucoside-5-*O*-(malonyl)glucoside isomer 2	[M]^+^	11.62	1211.3	1167.4 [M-CO_2_]^+^; 963.3 [M-Hex-Mal]^+^; 535.2 [M-2Hex-Cou-Sin]^+^; 491.1 [M-2Hex-Cou-Sin-CO_2_]^+^
Isorhamnetin 3-*O*-sinapoylglucoside-7-*O*-glucoside	[M-H]^−^	11.69	845.3	827.2 [M-H-H_2_O]^−^; 683.2 [M-H-Hex]^−^; 477.2 [M-H-Hex-Sin]^−^; 315.2 [M-H-2Hex-Sin]^−^
Quercetin 3-*O*-feruloylsophorotrioside-7-*O*-glucoside	[M-H]^−^	11.77	1125.3	963.2 [M-H-Hex]^−^; 949.3 [M-H-Fer]^−^; 787.0 [M-H-Hex-Fer]^−^
Diferuloylgentibiose	[M-H]^−^	12.00	693.1	675.1 [M-H-H_2_O]^−^; 517.2 [M-H-Fer]^−^; 531.3 [M-H-Hex]^−^; 499.3 [M-H-Fer-H_2_O]^−^; 355.2 [M-H-Hex-Fer]^−^
Trisinapoylgentiobiose	[M-H]^−^	13.22	959.2	753.2 [M-H-Sin]^−^; 735.2 [M-H-Sin-H_2_O]^−^; 591.2 [M-H-Sin-Hex]^−^; 529.3 [M-H-2Sin-H_2_O]^−^; 511.2 [M-H-2Sin-2H_2_O]^−^
Feruloyl-disinapoyl-gentiobiose	[M-H]^−^	13.42	929.2	753.1 [M-H-Fer]^−^; 723.2 [M-H-Sin]^−^; 705.4 [M-H-Sin-H_2_O]^−^; 529.3 [M-H-Sin-Fer-H_2_O]^−^; 511.2 [M-H-Sin-Fer-2H_2_O]^−^; 323.1 [M-H-2Sin-Fer-H_2_O]^−^

Abbreviations: Hex, Rha, Sin, Fer, HFer, Cou, Caf, Mal indicate hexoside, rhamnoside, sinapoyl, feruloyl, hydroxyferuloyl coumaroyl, caffeoyl, malonyl moieties, respectively.

⁎ The identification of this molecule was confirmed using a commercial standard.

### 3.5 Spectrophotometric determination of total polyphenol content (TPC) and total anthocyanins content (TAC)

After defining the OM qualitative composition in terms of GSLs and polyphenols, the subsequent step was to evaluate its overall quantitative profile, with a secondary particular focus on total polyphenol and anthocyanin contents, as these compounds may also contribute to the nutraceutical potential of the final product. For this purpose, the total concentration of polyphenols was determined spectrophotometrically using the Folin–Ciocalteu assay, while the total amount of anthocyanins was quantified by differential absorption methods (Table 4). Our results highlight that the TPC reached 17.21 ± 0.67 mg GAE/g DW**,** while the TAC was 11.91 ± 0.02 mg C3G/g DW in the OM sample. These results indicate that anthocyanins contribute to more than 50% of the total phenolic content, highlighting their predominant role in shaping the phenolic profile of the microgreens sample. Our findings are in line with those reported by (Dhaka et al., 2023); who observed valuable anthocyanin concentrations in red radish microgreens of 186 mg/100 g fresh weight. Similarly; Gunjal et al. (2024) quantified 27.94–28.25 μmol/100 g in fresh weight in ‘Sango’ radish microgreens (Gunjal et al., 2024). These results indicate that anthocyanins represent a valuable proportion of the phenolic fraction in pigmented radish microgreens, consistent with their predominant contribution observed in OM.

**Table 4:** Total polyphenol content and total anthocyanin content of OM.

Total polyphenols content (mg GAE/g DW ± SD)	TAC (mg C3G/g DW)
17.21 ± 0.67	11.91 ± 0.02

The results are expressed as mmol TE per gram of DW OM. Abbreviations: OM, Optimized Microgreen; DPPH, 2,2-diphenyl-1-picrylhydrazyl; ABTS, 2,2-azino-bis (3-ethylbenzothiazoline-6-sulfonic acid); FRAP, ferric reducing antioxidant power; TE, Trolox equivalent. Values are mean ± standard deviation (SD) of three repetitions (*n* = 3).

### 3.6 Antioxidant activity

**Fig. 1:**
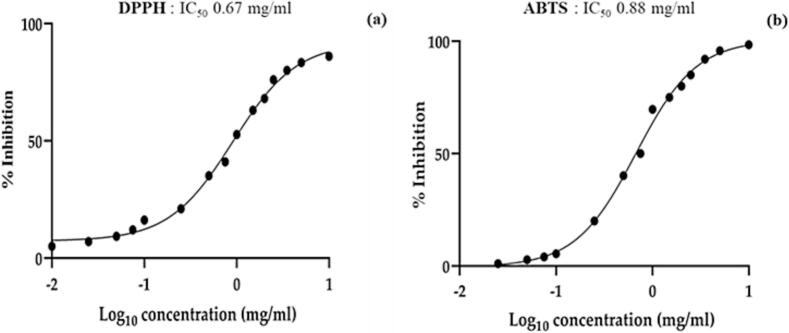
Antiradical potential of OM expressed as EC_50_ of DPPH assay (a) and EC_50_ of ABTS assay (b). Values represent the mean ± standard deviation of triplicate analysis (*n* = 3).

To further explore the biofunctional profile of the optimized microgreens, their antioxidant activity was evaluated using three complementary *in vitro* spectrophotometric assays: ferric reducing antioxidant power (FRAP), 2,2′-azino-bis-(3-ethylbenzothiazoline-6-sulfonic acid) (ABTS), and 2,2-diphenyl-1-picrylhydrazyl (DPPH). As shown in Table 5, the FRAP assay yielded the highest antioxidant capacity, with a calculated value of 95.16 ± 2.55 μmol TE/g DW. The ABTS and DPPH assays also confirmed the antioxidant potential of the microgreens, with results of 72.33 ± 0.03 and 44.01 ± 2.25 μmol TE/g DW, respectively. To standardize comparison across the different antioxidant assays, the results of the DPPH and ABTS tests were also expressed as EC_50_ values, representing the amount of antioxidant required to reduce the initial radical concentration by 50%. Although all three assays are based on the electron transfer (ET) mechanism, they differ in their sensitivity to different classes of antioxidants and reaction conditions. The calculated EC_50_ values were 0.67 mg/mL for the DPPH assay and 0.88 mg/mL for the ABTS assay (Fig. 1), further confirming the marked OME radical-scavenging capacity. The consistent response observed across all methods used emphasises the robustness and reliability of the antiradical profile of the prepared microgreen extract. This result also supports the hypothesis that the high antiradical potential is mainly related to the high content of polyphenols and anthocyanins, which are well-known effective electron donors in redox systems. Such pronounced antiradical activity is particularly relevant in the context of vascular alternation, as oxidative stress is a well-recognized driver of endothelial dysfunction and cardiovascular (Förstermann et al., 2017; Sena et al., 2018), thereby suggesting that the antioxidant profile of OME may potentiate the vasculoprotective effects conferred by glucosinolate-derived H₂S release. However, a limitation of the present study is that the antioxidant activity was evaluated only using *in vitro* chemical assays (DPPH, ABTS, and FRAP), which provide an estimation of radical scavenging capacity but do not necessarily translate into biological antioxidant effects *in vivo*, which will require further biological investigations.

**Table 5:** Antioxidant activity of OM microgreens evaluated by DPPH, ABTS, and FRAP assays.

Antioxidant Activity (μmol TE/g DW ± SD)			
	FRAP assay	ABTS assay	DPPH assay
OM microgreens	95.16 ± 2.55	72.33 ± 0.03	44.01 ± 2.25

The results are expressed as mmol TE per gram of microgreen sample. Abbreviations: DPPH, 2,2-diphenyl-1-picrylhydrazyl; ABTS, 2,2′-azino-bis (3-ethylbenzothiazoline-6-sulfonic acid); FRAP, ferric reducing antioxidant power; TE, Trolox equivalent. Values are mean ± standard deviation (SD) of three repetitions (n = 3).

### 3.7 Hydrogen sulfide release

**Fig. 2:**
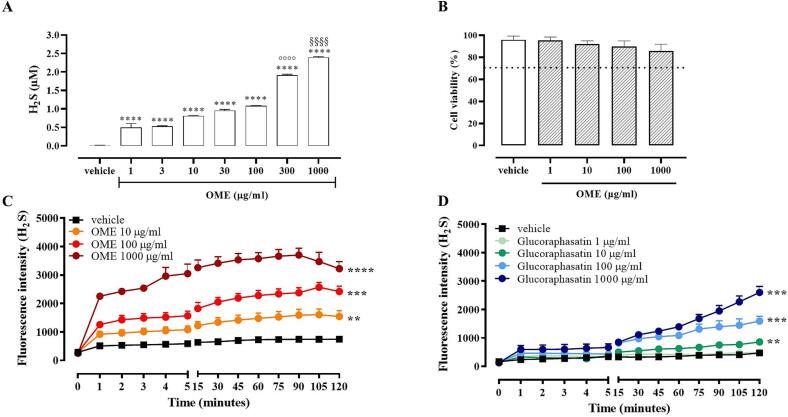
A) H_2_S measurement from OME (1–1000 μg/mL) in a cell-free assay. Data are reported as μM and expressed as means ± SEM (n = 3; **** *p* < 0.0001 *vs* vehicle; °°°° *p* < 0.0001 *vs* 100 μg/mL, and §§§§ *p* < 0.0001 *vs* 300 μg/mL); B) MTT assay for OME (1–1000 μg/mL) in BAEC C) C) Live fluorometric quantification of H_2_S in BAEC exposed to OME (10–1000 μg/mL) or glucoraphasatin (1–1000 μg/mL). Data are reported as fluorescence intensity and expressed as means ± SEM (*n* = 5; ** *p* < 0.01, *** *p* < 0.001, and **** *p* < 0.0001 *vs* vehicle).

**Fig. 3:**
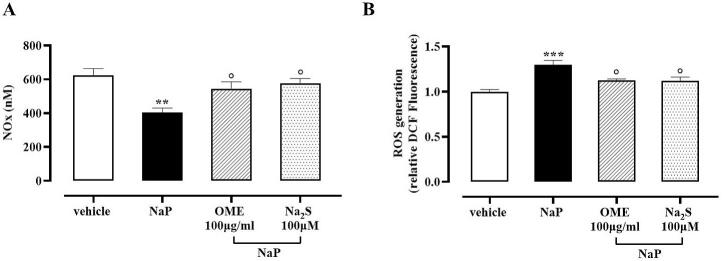
A) NO measurement (*n* = 4; ** *p* < 0.01 *vs* vehicle, ° *p* < 0.05 *vs* NaP) and B) ROS measurement (*** *p* < 0.001 *vs* vehicle, ° *p* < 0.05 *vs* NaP) in the supernatant of BAEC stimulated with NaP (100 μM, 4 h) and pretreated with vehicle, OME 100 μg/mL, or Na_2_S (100 μM) 30 min before challenge. Data are reported as nM and relative DCF fluorescence, respectively, and expressed as means ± SEM.

To evaluate OME's nutraceutical potential as H₂S donor, its capacity to release H₂S was first assessed in a cell-free system and subsequently confirmed in BAECs. Our results indicated that OME (1–1000 μg/mL) was able to release H_2_S in a cell-free assay compared to the vehicle. In detail, a significant increase in H_2_S release was found at all concentrations tested compared to vehicle, reaching values of about 2.5 μM at the highest concentration (**** *p* < 0.0001 *vs* vehicle; °°°° *p* < 0.0001 *vs* 100 μg/mL; §§§§ *p* < 0.0001 *vs* 300 μg/mL, Fig. 2A). To better define the relevance of OME as a source of H_2_S, we moved to BAEC. Therefore, cell viability following exposure to OME (1, 10, 100, and 1000 μg/mL) was assessed. Under these experimental conditions, OME did not alter cell viability, ruling out cytotoxic effects (Fig. 2B). Interestingly, a concentration-dependent increase in H_2_S was observed following the exposure of OME (10 μg/mL, 100 μg/mL and 1000 μg/mL) in BAEC (Fig. 2C, ** *p* < 0.01, *** *p* < 0.001, and **** *p* < 0.0001 *vs* vehicle) that persisted up to 120 min at all concentrations tested. Collectively, these results indicate OME as a naturally occurring source of H_2_S. Therefore, OME enters the class of “sulfanutraceutics”, which refers to vegetable extracts or edible plants containing sulfaeutics and acting as a source of exogenous H_2_S (Martelli et al., 2023b). As reported in Table 1; OME contains glucoraphasatin has been defined as slow donor of H_2_S; belonging to the class of the “sulfaceutics” (Gambari et al., 2022; Martelli et al., 2023b; Micheli et al., 2022). Specifically, glucoraphasatin represents the most abundant GSL in OME. Thus, we evaluated the ability of glucoraphasatin (1, 10, 100, and 1000 μg/mL) to release H_2_S in BAEC. Similarly to OME, glucoraphasatin released H_2_S in a concentration-dependent manner in BAEC up 120 min (Fig. 2D, ** *p* < 0.01 and *** *p* < 0.001 *vs* vehicle). Based on these results, we can classify an additional compound within the “sulfaceutic” category. The differing H₂S-release kinetics of OME and glucoraphasatin arise from the fact that OME is a complex extract containing multiple components that act synergistically, whereas glucoraphasatin is a pure active compound. H_2_S is well known for its vasodilatory properties, which improve blood flow and reduce blood pressure, making it a promising therapeutic agent (Munteanu et al., 2021). The therapeutic potential of H_2_S as a vasodilator is supported by a growing body of evidence from *in vitro* studies; preclinical animal models; and clinical trials. Specifically; the blood pressure-lowering efficacy of H₂S has been well documented; along with its ability to prevent vascular dysfunction (d'Emmanuele di Villa Bianca et al., 2015; Gorica et al., 2025; Sun et al., 2024; Yang et al., 2022). Recently, it has been shown that the isothiocyanate Erucin is a natural H_2_S-donor and improves the vasorelaxing response of human microvessels from patients with obesity to acetylcholine, in part through an improvement of the endothelial function and the modulation of SIRT1 expression, reducing mitochondrial oxidative stress (Gorica et al., 2025). In this scenario; the ability of OME to release H_2_S in a controlled and prolonged fashion makes it of particular interest in cardiovascular disorders. Indeed; we have tested the beneficial effect of OME by an *in vitro* model of lipid-overload associated with endothelial dysfunction in BAEC using a well-known H₂S donor (Smimmo et al., 2024), Na₂S, as a reference compound. OME treatment restores the increase in NO levels induced by NaP to the control condition (Fig. 3A, ** *p* < 0.01 *vs* vehicle, ° *p* < 0.05 *vs* NaP) and concurrently reduces ROS production (Fig. 3B, *** *p* < 0.001 *vs* vehicle, ° *p* < 0.05 *vs* NaP), showing a similar profile of Na_2_S. Overall, these results support the potential of OME as a promising candidate for mitigating oxidative stress and improving endothelial function under lipid-overload conditions. Certainly, the protective effects observed may be related to the contribution of these GLS-derived sulfur compounds, likely acting synergistically with other components present in the extract. While the H₂S-releasing glucosinolates are likely the main determinants of the observed effects, the contribution of the antioxidant polyphenolic fraction should also be considered, suggesting a possible cooperative action on oxidative stress modulation and endothelial redox homeostasis. This designation not only underscores the nutritional and pharmacological relevance of OME but also provides a foundation for further investigations and potential applications in pathological conditions characterized by dysregulated NO/H₂S signaling.

To the best of our knowledge, this is the first study demonstrating that an extract obtained from a sustainable and highly standardized microgreen source is not only able to release H₂S in endothelial cells, but also directly modulate and rebalance an altered cellular redox state under oxidative stress–prone conditions. Thus, beyond the phytochemical characterization of glucosinolate-rich microgreens, the present work provides functional evidence that OME acts as a biologically active sulfanutraceutical matrix, with potential application in management of cardiovascular diseases.

## 4 Conclusion

This study highlights the potential of *Raphanus sativus* cv. Tango microgreens as a highly standardized and versatile source of bioactive compounds with remarkable nutraceutical relevance. To this end, different growth times and cultivation conditions were investigated in order to maximize GSLs accumulation. Analytical validation of the HPLC-qQq-MS/MS method allowed accurate quantification of intact GSLs, with glucoraphasatin emerging as the predominant compound. Overall, the total GSLs content at the optimal harvesting time (T2, 6 days) reached 70.71 mg of GSL/g DW. Beyond their role as dietary precursors of H₂S, the microgreens were also characterized for their antioxidant potential, a property of particular interest for cardiovascular protection. At the optimal harvesting time, the total polyphenol content (TPC) was 17.21 ± 0.67 mg GAE/g DW, while anthocyanins (TAC) accounted for more than half of the phenolic pool (11.91 ± 0.02 mg C3G/g DW). This composition is responsible for the consistent antioxidant profile detected across multiple assays: FRAP 95.16 ± 2.55 μmol TE/g DW, ABTS 72.33 ± 0.03 μmol TE/g DW, and DPPH 44.01 ± 2.25 μmol TE/g DW. Additionally, the OME exhibited a valuable ability to release H₂S. In a cell-free system, OME triggered a concentration-dependent H₂S release up to 2.5 μM at 1000 μg/mL (*p* < 0.0001 *vs* vehicle). This effect was corroborated by using BAECs, where OME exposure (10–1000 μg/mL) sustained intracellular H₂S production for up to 120 min (*p* < 0.001 *vs* vehicle). A similar profile was found for glucoraphastin, the most abundant GSL in OME, identifying it as a slow donor of H_2_S belonging to the category of “sulfaceutics”. ì Our findings qualify OME as a promising “sulfanutraceutical,” able to combine antioxidant and gasotransmitter-releasing properties in a single source. Taken together, these results emphasize the dual nutraceutical potential of radish microgreens: (i) as a rich source of polyphenols and anthocyanins conferring high antioxidant capacity, and (ii) as a natural dietary donor of H₂S, with promising implications for cardiovascular health. Nevertheless, further experiments in *ex vivo* and *in vivo* models, as well as investigations aimed at elucidating the underlying molecular mechanisms, are required to confirm and extend these findings.

## CRediT authorship contribution statement

**Maria Maisto:** Writing – original draft, Visualization, Supervision, Software, Methodology, Investigation, Formal analysis, Data curation, Conceptualization. **Adua Marzocchi:** Writing – original draft, Software, Methodology, Investigation, Formal analysis, Data curation, Conceptualization. **Vincenzo Piccolo:** Writing – original draft, Visualization, Software, Methodology, Investigation, Formal analysis, Data curation, Conceptualization. **Erika Esposito:** Writing – original draft, Software, Methodology, Investigation, Formal analysis, Data curation. **Melania Correale:** Methodology, Investigation, Formal analysis, Data curation. **Emma Mitidieri:** Writing – review & editing, Visualization, Supervision, Methodology, Investigation. **Gian Carlo Tenore:** Writing – review & editing, Visualization, Supervision, Resources, Project administration, Funding acquisition. **Roberta d'Emmanuele di Villa Bianca:** Writing – review & editing, Visualization, Supervision, Project administration.

## Funding source

This research did not receive any specific grant from funding agencies in the public, commercial, or not-for-profit sectors.

## Declaration of competing interest

The authors declare that they have no known competing financial interests or personal relationships that could have appeared to influence the work reported in this paper.

## Data Availability

Data will be made available on request.

## References

[bb0005] Abe K., Kimura H. (1996). The possible role of hydrogen sulfide as an endogenous neuromodulator. The Journal of Neuroscience.

[bb0010] Bello I., Smimmo M., d’Emmanuele di Villa Bianca R., Bucci M., Cirino G., Panza E., Brancaleone V. (2023). Erucin, an H2S-releasing isothiocyanate, exerts anticancer effects in human triple-negative breast cancer cells triggering autophagy-dependent apoptotic cell death. International Journal of Molecular Sciences.

[bb0015] Bellostas N., Kachlicki P., Sørensen J.C., Sørensen H. (2007). Glucosinolate profiling of seeds and sprouts of *B. oleracea* varieties used for food. Scientia Horticulturae.

[bb0020] Bones A.M., Rossiter J.T. (1996). The myrosinase-glucosinolate system, its organisation and biochemistry. Physiologia Plantarum.

[bb0025] Casertano M., Esposito E., Bello I., Indolfi C., Putra M., Di Cesare Mannelli L., Mitidieri E. (2023). Searching for novel sources of hydrogen sulfide donors: Chemical profiling of *Polycarpa aurata* extract and evaluation of the anti-inflammatory effects. Marine Drugs.

[bb0030] Cirino G., Szabo C., Papapetropoulos A. (2023). Physiological roles of hydrogen sulfide in mammalian cells, tissues, and organs. Physiological Reviews.

[bb0035] Citi V., Martelli A., Gorica E., Brogi S., Testai L., Calderone V. (2021). Role of hydrogen sulfide in endothelial dysfunction: Pathophysiology and therapeutic approaches. Journal of Advanced Research.

[bb0040] Demir K., Sarıkamış G., Çakırer Seyrek G. (2023). Effect of LED lights on the growth, nutritional quality and glucosinolate content of broccoli, cabbage and radish microgreens. Food Chemistry.

[bb0045] d’Emmanuele di Villa Bianca R., Mitidieri E., Donnarumma E., Tramontano T., Brancaleone V., Cirino G., Sorrentino R. (2015). Hydrogen sulfide is involved in dexamethasone-induced hypertension in rat. Nitric Oxide.

[bb0050] Dhaka A.S., Dikshit H.K., Mishra G.P., Tontang M.T., Meena N.L., Kumar R.R., Praveen S. (2023). Evaluation of growth conditions, antioxidant potential, and sensory attributes of six diverse microgreens species. Agriculture.

[bb0055] Di Lorenzo R., Bernardi A., Grumetto L., Sacchi A., Avagliano C., Coppola S., Dini I. (2021). Phenylalanine butyramide is a new cosmetic ingredient with soothing and anti-reddening potential. Molecules.

[bb0060] Di Lorenzo R., Castaldo L., Sessa R., Ricci L., Vardaro E., Izzo L., Grosso M., Ritieni A., Laneri S. (2024). Chemical profile and promising applications of *Cucurbita pepo* L. flowers. Antioxidants.

[bb0065] Esposito E., Indolfi C., Bello I., Smimmo M., Vellecco V., Schettino A., Mitidieri E. (2024). The endocrine disruptor vinclozolin causes endothelial injury via eNOS/Nox4/IRE1α signaling. European Journal of Pharmacology.

[bb0070] Fayezizadeh M.R., Ansari N.A., Sourestani M.M., Fujita M., Hasanuzzaman M. (2024). Management of secondary metabolite synthesis and biomass in basil (*Ocimum basilicum* L.) microgreens using different continuous-spectrum LED lights. Plants.

[bb0075] Förstermann U., Xia N., Li H. (2017). Roles of vascular oxidative stress and nitric oxide in the pathogenesis of atherosclerosis. Circulation Research.

[bb0080] Galieni A., Falcinelli B., Stagnari F., Datti A., Benincasa P. (2020). Sprouts and microgreens: Trends, opportunities, and horizons for novel research. Agronomy.

[bb0085] Gallaher C.M., Gallaher D.D., Peterson S. (2012). Development and validation of a spectrophotometric method for quantification of total glucosinolates in cruciferous vegetables. Journal of Agricultural and Food Chemistry.

[bb0090] Gambari L., Barone M., Amore E., Grigolo B., Filardo G., Iori R., Citi V., Calderone V., Grassi F. (2022). Glucoraphanin increases intracellular hydrogen sulfide (H2 S) levels and stimulates osteogenic differentiation in human mesenchymal stromal cell. Nutrients.

[bb0095] Gorica E., Piragine E., Mengozzi A., Cappelli F., Duranti E., Masi S., Virdis A., Testai L., Pagnotta E., Righetti L., Costantino S., Paneni F., Calderone V., Martelli A. (2025). The H2S-donor Erucin modulates SIRT1 and rescues obesity-induced vascular dysfunction in human vessels. Biomedicine & Pharmacotherapy.

[bb0100] Gunjal M., Singh J., Kaur S., Nanda V., Ullah R., Iqbal Z., Ercisli S., Rasane P. (2024). Assessment of bioactive compounds, antioxidant properties and morphological parameters in selected microgreens cultivated in soilless media. Scientific Reports.

[bb0105] Huseby S., Koprivova A., Lee B.-R., Saha S., Mithen R., Wold A.-B., Kopriva S. (2013). Diurnal and light regulation of sulphur assimilation and glucosinolate biosynthesis in Arabidopsis. Journal of Experimental Botany.

[bb0110] Iannuzzo F., Piccolo V., Novellino E., Schiano E., Salviati E., Summa V., Maisto M. (2022). A Food-grade method for enhancing the levels of low molecular weight proanthocyanidins with potentially high intestinal bioavailability. International Journal of Molecular Sciences.

[bb0115] Jain S.K., Bull R., Rains J.L., Bass P.F., Levine S.N., Reddy S., Bocchini J.A. (2010). Low levels of hydrogen sulfide in the blood of diabetes patients and streptozotocin-treated rats causes vascular inflammation?. Antioxidants & Redox Signaling.

[bb0120] Jauregui M.J., Warren E.R., Di Gioia F., Kwasniewski M.T., Lambert J.D. (2025). Effects of hot air drying on the nutritional and phytochemical composition of radish (*Raphanus sativus* L.) microgreens. Journal of Food Science.

[bb0125] Laezza C., Imbimbo P., D’Amelia V., Marzocchi A., Monti D.M., Di Loria A., Rigano M.M. (2024). Use of yeast extract to elicit a pulp-derived callus cultures from Annurca apple and potentiate its biological activity. Journal of Functional Foods.

[bb0130] Liu Z., Shi J., Wan J., Pham Q., Zhang Z., Sun J., Chen P. (2022). Profiling of polyphenols and glucosinolates in kale and broccoli microgreens grown under chamber and windowsill conditions by ultrahigh-performance liquid chromatography high-resolution mass spectrometry. ACS Food Science & Technology.

[bb0135] López-Belchí M.D., Toro M.T., Illanes M., Henríquez-Aedo K., Fernández-Martinez J., Schoebitz M., Moreno D.A. (2024). Exploring strategies to growth wild turnip sprouts as healthy food. Chemical and Biological Technologies in Agriculture.

[bb0140] Maisto M., Piccolo V., Novellino E., Schiano E., Iannuzzo F., Ciampaglia R., Tenore G.C. (2023). Optimization of ursolic acid extraction in oil from annurca apple to obtain oleolytes with potential cosmeceutical application. Antioxidants.

[bb0145] Martelli A., Citi V., Testai L., Brogi S., Calderone V. (2020). Organic isothiocyanates as hydrogen sulfide donors. Antioxidants & Redox Signaling.

[bb0150] Martelli A., d’Emmanuele di Villa Bianca R., Cirino G., Sorrentino R., Calderone V., Bucci M. (2023). Hydrogen sulfide and sulfaceutic or sulfanutraceutic agents: Classification, differences and relevance in preclinical and clinical studies. Pharmacological Research.

[bb0155] Martelli A., d’Emmanuele di Villa Bianca R., Cirino G., Sorrentino R., Calderone V., Bucci M. (2023). Hydrogen sulfide and sulfaceutic or sulfanutraceutic agents: Classification, differences and relevance in preclinical and clinical studies. Pharmacological Research.

[bb0160] Michalczyk M. (2025). Methods of modifying the content of glucosinolates and their derivatives in sprouts and microgreens during their cultivation and postharvest handling. International Journal of Food Science.

[bb0165] Micheli L., Mitidieri E., Turnaturi C., Vanacore D., Ciampi C., Lucarini E., Di Villa Bianca R.D. (2022). Beneficial effect of H_2_S-releasing molecules in an in vitro model of sarcopenia: Relevance of glucoraphanin. International Journal of Molecular Sciences.

[bb0170] Mitidieri E., Vanacore D., Turnaturi C., Sorrentino R., d’Emmanuele di Villa Bianca R. (2020). Uterine dysfunction in diabetic mice: The role of hydrogen sulfide. Antioxidants.

[bb0175] Munteanu C., Munteanu D., Onose G. (2021). Hydrogen sulfide (H2S) - therapeutic relevance in rehabilitation and balneotherapy systematic literature review and meta-analysis based on the PRISMA paradig. Balneo and PRM Research Journal.

[bb0180] Naresh R., Jadav S.K., Singh M., Patel A., Singh B., Beese S., Pandey S.K. (2024). Role of hydroponics in improving water-use efficiency and food security. International journal of environment and climate change.

[bb0185] Ohno Y. (2002). ICH guidelines—Implementation of the 3Rs (refinement, reduction, and replacement): Incorporating best scientific practices into the regulatory process. ILAR Journal.

[bb0190] Olivencia M.A., Esposito E., Brancaleone V., Castaldo S., Cirino G., Pérez-Vizcaino F., Mitidieri E. (2023). Hydrogen sulfide regulates the redox state of soluble guanylate cyclase in CSE−/− mice corpus cavernosum microcirculation. Pharmacological Research.

[bb0195] Pardini A., Tamasi G., De Rocco F., Bonechi C., Consumi M., Leone G., Rossi C. (2021). Kinetics of glucosinolate hydrolysis by myrosinase in brassicaceae tissues: A high-performance liquid chromatography approach. Food Chemistry.

[bb0200] Piccolo V., Maisto M., Cerrato L.M., Esposito E., Panza E., Sorrentino R., Summa V. (2024). Characterization and quantification of intact glucosinolates in catozza rapeseeds: A promising food matrix for nutraceuticals development as a source of hydrogen sulfide. Journal of Functional Foods.

[bb0205] Piccolo V., Maisto M., Schiano E., Iannuzzo F., Keivani N., Manuela Rigano M., Santini A., Novellino E., Carlo Tenore G., Summa V. (2024). Phytochemical investigation and antioxidant properties of unripe tomato cultivars (*Solanum lycopersicum* L.). Food Chemistry.

[bb0210] Piragine E., Malanima M.A., Lucenteforte E., Martelli A., Calderone V. (2023). Circulating levels of hydrogen sulfide (H2S) in patients with age-related diseases: A systematic review and meta-analysis. Biomolecules.

[bb0215] Sena C.M., Leandro A., Azul L., Seiça R., Perry G. (2018). Vascular oxidative stress: Impact and therapeutic approaches. Frontiers in Physiology.

[bb0220] Smimmo M., Casale V., Casillo G.M., Mitidieri E., d’Emmanuele di Villa Bianca R., Bello I., Vellecco V. (2024). Hydrogen sulfide dysfunction in metabolic syndrome-associated vascular complications involves cGMP regulation through soluble guanylyl cyclase persulfidation. Biomedicine & Pharmacotherapy.

[bb0225] Sun X., Wu S., Mao C., Qu Y., Xu Z., Xie Y., Song Y. (2024). Therapeutic potential of hydrogen sulfide in ischemia and reperfusion injury. Biomolecules.

[bb0230] Swer T.L., Chauhan K., Paul P.K., Mukhim C. (2016). Evaluation of enzyme treatment conditions on extraction of anthocyanins from *Prunus nepalensis* L. International Journal of Biological Macromolecules.

[bb0235] Wang J., Mao S., Wu Q., Yuan Y., Liang M., Wang S., Huang K., Wu Q. (2021). Effects of LED illumination spectra on glucosinolate and sulforaphane accumulation in broccoli seedlings. Food Chemistry.

[bb0240] Yang Y.-W., Deng N.-H., Tian K.-J., Liu L.-S., Wang Z., Wei D.-H., Liu H.-T., Jiang Z.-S. (2022). Development of hydrogen sulfide donors for anti-atherosclerosis therapeutics research: Challenges and future priorities. Frontiers in Cardiovascular Medicine.

[bb0245] Yarmohammadi F., Hayes A.W., Karimi G. (2021). The cardioprotective effects of hydrogen sulfide by targeting endoplasmic reticulum stress and the Nrf2 signaling pathway: A review. BioFactors.

[bb0250] Ying Q., Kong Y., Zheng Y. (2020). Applying blue light alone, or in combination with far-red light, during nighttime increases elongation without compromising yield and quality of indoor-grown microgreens. HortScience.

[bb0255] Zhang H., Pan J., Huang S., Chen X., Chang A.C.Y., Wang C., Zhang H. (2024). Hydrogen sulfide protects cardiomyocytes from doxorubicin-induced ferroptosis through the SLC7A11/GSH/GPx4 pathway by Keap1 S-sulfhydration and Nrf2 activation. Redox Biology.

[bb0260] Zhou B., Feng X., Huang W., Liu Q., Ibrahim S.A., Liu Y. (2023). Effects of light intensity on the biosynthesis of glucosinolate in Chinese cabbage plantlets. Scientia Horticulturae.

