# Supplementary material for: Nutraceutical potential of radish (Raphanus sativus cv. Tango) microgreens as sustainable and reproducible sources of hydrogen sulfide-releasing compounds
Source: Food Chem X. 2026 Apr 2;35:103816. doi: 10.1016/j.fochx.2026.103816 (PMC13090313; doi:10.1016/j.fochx.2026.103816)

**Nutraceutical Potential of Radish (*Raphanus sativus* cv. Tango) Microgreens as Sustainable and Reproducible Sources of Hydrogen Sulfide-Releasing Compounds**

Maria Maisto^1†^, Adua Marzocchi^1†^, Vincenzo Piccolo^1^*, Erika Esposito^2^, Melania Correale^2^, Emma Mitidieri^2^, Gian Carlo Tenore^1^, and Roberta d’Emmanuele di Villa Bianca^2^

1. Nutrapharmalab, Department of Pharmacy, School of Medicine and Surgery, University of Napoli Federico II, Via Domenico Montesano 49, 80131, Napoli, Italy
2. Department of Pharmacy, School of Medicine and Surgery, University of Napoli Federico II, Via Domenico Montesano 49, 80131, Napoli, Italy

* Corresponding author: Vincenzo Piccolo; vincenzo.piccolo3@unina.it; Tel.: +39-389-205-37-63

† These authors contributed equally to this work

Table S1. MRM parameters for GSLs quantification by HPLC-MS/MS, including retention times, precursor ions, fragmentor voltages, and collision energies.

| Compound | Rt | Precursor ion | Fragmentor | Quantifier (CID) | Qualifier (CID) |
| --- | --- | --- | --- | --- | --- |
| Glucoraphanin | 2.90 | 436.3 | 146 | 96.0 (80) | 372.1 (24) |
| Glucoraphenin | 3.10 | 434.4 | 182 | 97.0 (28) | 96.0 (32) |
| Glucoraphasatin | 7.20 | 418.3 | 182 | 97.0 (24) | 96.0 (48) |

Table S2. Analytical performance parameters of the HPLC-MS/MS method, including linearity range, sensitivity (LOD and LOQ), quality control analytical stability (RSD%), and carry-over assessment for monitored GSLs.

| Compound | Linearity range (ppm) | Calibration curve | R2 | LOD (ppb) | LOQ (ppb) | QC (RSD% %) | Carry-over % |
| --- | --- | --- | --- | --- | --- | --- | --- |
| Glucoraphanin | 0.0025 – 5 | y=4334.9x-33.683 | 0.9999 | 0.68 ± 0.40 | 2.06 ± 1.20 | 3.91 | 1.50*10^-4^ |
| Glucoraphenin | 0.0025 – 10 | y=8370.4x-0.9496 | 0.9999 | 0.53 ± 0.19 | 1.61 ± 0.59 | 3.79 | 8.09*10^-7^ |
| Glucoraphasatin | 0.010 – 5 | y=13734x+17.657 | 0.9999 | 0.25 ± 0.01 | 0.76 ± 0.03 | 3.90 | 3.50*10^-5^ |

Table S3. Precision and accuracy values of the HPLC MS/MS method for monitored GSLs.

| Compound | Concentration (ppm) | Precision (CV%) | | Accuracy (% bias) | |
| --- | --- | --- | --- | --- | --- |
|  |  | Intraday | Interday | Intraday | Interday |
| Glucoraphanin | 5 | 1.00 | 2.72 | 1.35 | -3.93 |
|  | 0.155 | 1.41 | 1.98 | 0.54 | 0.49 |
|  | 0.0025 | 8.80 | 8.85 | 0.81 | 0.79 |
| Glucoraphenin | 10 | 0.42 | 1.66 | -0.50 | -8.61 |
|  | 0.155 | 1.98 | 1.67 | 0.29 | 0.55 |
|  | 0.0025 | 7.39 | 8.51 | 0.05 | -0.01 |
| Glucoraphasatin | 5 | 0.88 | 3.73 | 0.24 | -0.15 |
|  | 0.155 | 0.62 | 1.72 | 0.90 | 0.70 |
|  | 0.010 | 4.17 | 3.47 | -0.13 | -0.16 |

Figure S1. Evaluation of temperature-dependent myrosinase activity using glucoraphasatin as substrate.


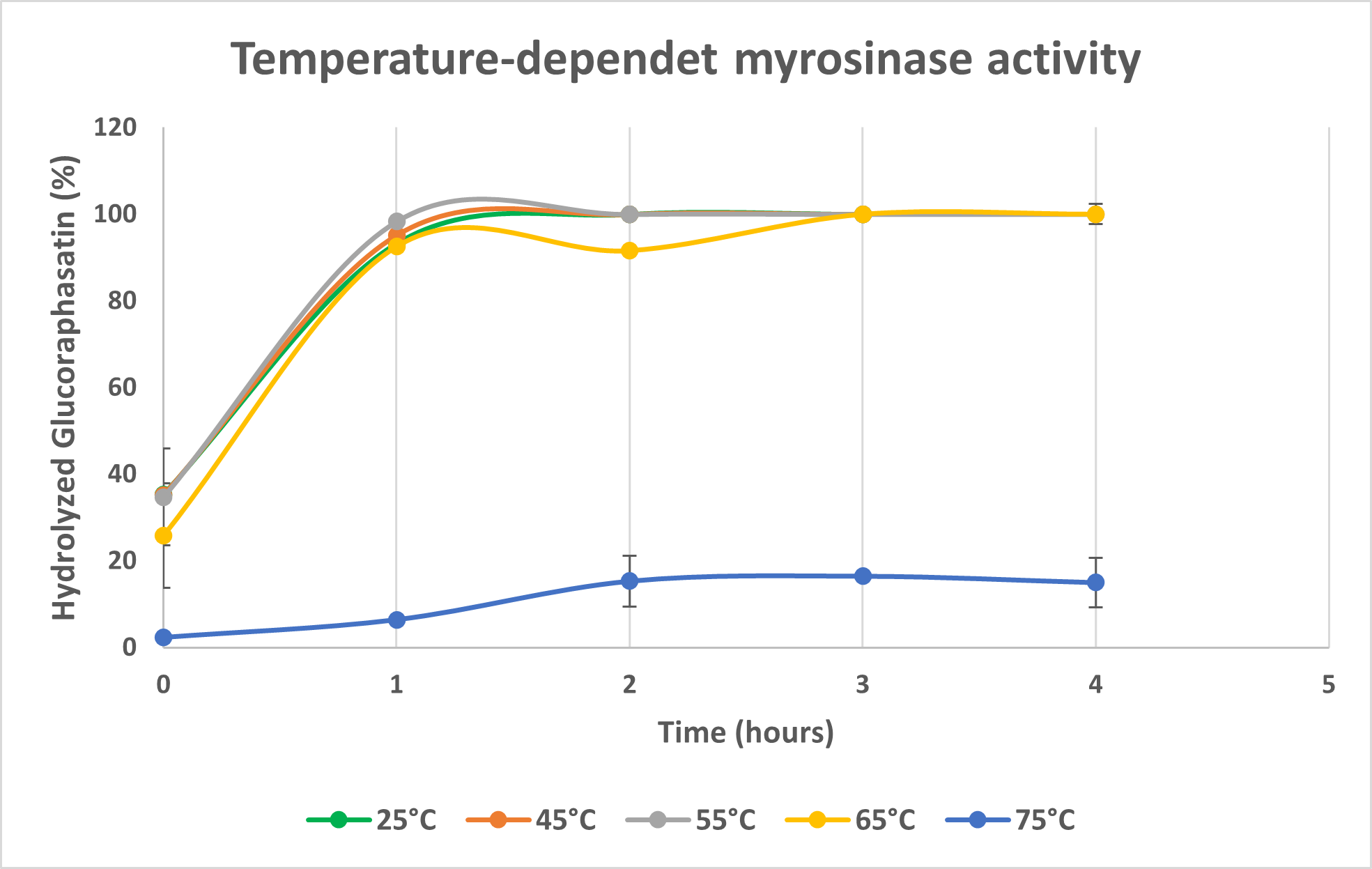

Supplement: Supplementary file 1 — Supplementary material. [file mmc1.docx]
